# Neurodevelopmental Trajectories in Children With Internalizing, Externalizing and Emotion Dysregulation Symptoms

**DOI:** 10.3389/fpsyt.2022.846201

**Published:** 2022-03-18

**Authors:** Elisabet Blok, Eloy P. T. Geenjaar, Eloïse A. W. Geenjaar, Vince D. Calhoun, Tonya White

**Affiliations:** ^1^Department of Child and Adolescent Psychiatry/Psychology, Erasmus MC Sophia Childrens Hospital, University Medical Center Rotterdam, Rotterdam, Netherlands; ^2^The Generation R Study Group, Erasmus MC, University Medical Centre Rotterdam, Rotterdam, Netherlands; ^3^School of Electrical and Computer Engineering, Georgia Institute of Technology, Atlanta, GA, United States; ^4^Tri-institutional Center for Translational Research in Neuroimaging and Data Science (TReNDS), Atlanta, GA, United States; ^5^Department of Radiology and Nuclear Medicine, Erasmus MC, University Medical Centre Rotterdam, Rotterdam, Netherlands

**Keywords:** neurodevelopment, normative modeling, childhood, adolescence, psychopathology

## Abstract

**Introduction:**

Childhood and adolescence are crucial periods for brain and behavioral development. However, it is not yet clear how and when deviations from typical brain development are related to broad domains of psychopathology.

**Methods:**

Using three waves of neuroimaging data within the population-based Generation R Study sample, spanning a total age range of 6–16 years, we applied normative modeling to establish typical development curves for (sub-)cortical volume in 37 brain regions, and cortical thickness in 32 brain regions. Z-scores representing deviations from typical development were extracted and related to internalizing, externalizing and dysregulation profile (DP) symptoms.

**Results:**

Normative modeling showed regional differences in developmental trajectories. Psychopathology symptoms were related to negative deviations from typical development for cortical volume in widespread regions of the cortex and subcortex, and to positive deviations from typical development for cortical thickness in the orbitofrontal, frontal pole, pericalcarine and posterior cingulate regions of the cortex.

**Discussion:**

Taken together, this study charts developmental curves across the cerebrum for (sub-)cortical volume and cortical thickness. Our findings show that psychopathology symptoms, are associated with widespread differences in brain development, in which those with DP symptoms are most heavily affected.

## 1. Introduction

Over the course of childhood and adolescence, both the brain and behavior undergo tremendous development. Regarding the relationship between the developing brain and atypical behavior, a body of evidence has associated differences in brain morphology to multiple domains of psychopathology (1–5). These studies have assessed multiple measures of brain morphology, including cortical volume and cortical thickness. However, the brain regions that have been identified are widespread and vary substantially across studies (5, 6). Additionally, the direction of effect also differs across studies, meaning that some studies find positive relationships between cortical thickness/volume and psychopathology, whereas others find negative associations (1, 7–10).

The age at which children are assessed may potentially be a crucial factor to unravel why effects across studies differ in both location and direction. Non-linear patterns in brain development across age may partially underlie differences in the direction of observed effects. Total brain volume, for example, increases until adolescence, where it reaches a plateau and starts to decline (11), whereas gray matter volume reaches this peak in early childhood (12). Additionally, evidence suggests that distinct brain lobes and regions within lobes, develop at their own pace (11, 13). The asynchronous development of regions and lobes may be an explanation for the effect differences observed in the brain regions involved in these studies. Recent work has therefore used data-driven normative modeling, a technique that can be used to derive typical development curves for brain morphology (14–16). Emerging evidence suggests that deviations from typical brain development, estimated using these normative models, improves prediction of psychopathology over predictions based on raw brain morphology measures (17).

Two broad domains of psychopathology, that have been widely studied in children, in relation to brain morphology include the internalizing domain (e.g., anxiety, depression) and the externalizing domain (e.g., aggressive behavior). A third domain is emotion dysregulation, which includes symptoms of both the internalizing and externalizing domain. Regions that were reported most consistently across studies for the internalizing domain include the orbitofrontal cortex (OFC) (1, 6, 8, 9, 18, 19), rostral middle frontal cortex (2, 3, 20), anterior cingulate cortex (ACC) (2, 3, 6, 19), amygdala (2, 3, 7, 20, 21) and hippocampus (2, 6, 18, 20). Regions that have shown to be associated with externalizing symptoms partially overlap with those reported for internalizing symptoms. These include the OFC (22), ACC (23–25), amygdala (4, 5, 26–28), hippocampus (24, 27, 29) and striatum (10, 23, 26). Research on emotion dysregulation is relatively scarce. However, Shaw et al. proposed that the OFC, amygdala and striatum, brain regions involved in the bottom-up response to emotional cues, are mainly associated with symptoms of emotion dysregulation (30).

The direction of the effect in earlier work on internalizing symptoms, seems to be dependent on the age range that is used in studies. Namely, studies including younger age ranges generally observed positive associations (1, 7, 19), whereas in older age ranges negative associations are observed (8, 9). In contrast, for externalizing symptoms, the majority of studies, including a meta-analysis for cortical and subcortical gray matter volume, point toward lower volume and thickness in children with externalizing disorders (5, 10, 22, 24–29), while a few report higher cortical volume or thickness (10, 31) and one reports a non-linear relationship (23).

The aims of this study were (i) to establish normative development curves for cortical thickness and (sub-)cortical volumes, covering the gray matter of the cerebrum, and (ii) to study to what extent deviations from typical development are related to psychopathology symptoms in a large population-based cohort of children and adolescents. We hypothesized that all three domains of psychopathology would be related to deviations from normative development of brain morphology. Specifically, we hypothesized that for internalizing symptoms, alterations would be most prominent in the rostral middle frontal cortex, OFC, amygdala and hippocampus; for externalizing in the ACC, OFC, amygdala, hippocampus and striatum; and for DP symptoms in the OFC, amygdala and striatum. Further, we hypothesized that the direction of these deviations varies with age for internalizing symptoms, with positive deviations at younger and negative deviations at older ages. For the externalizing domain we hypothesized that, in line with most prior work, higher symptoms are related to negative deviations from normative development at all ages.

## 2. Materials and Methods

### 2.1. Participants

**Figure 1:**
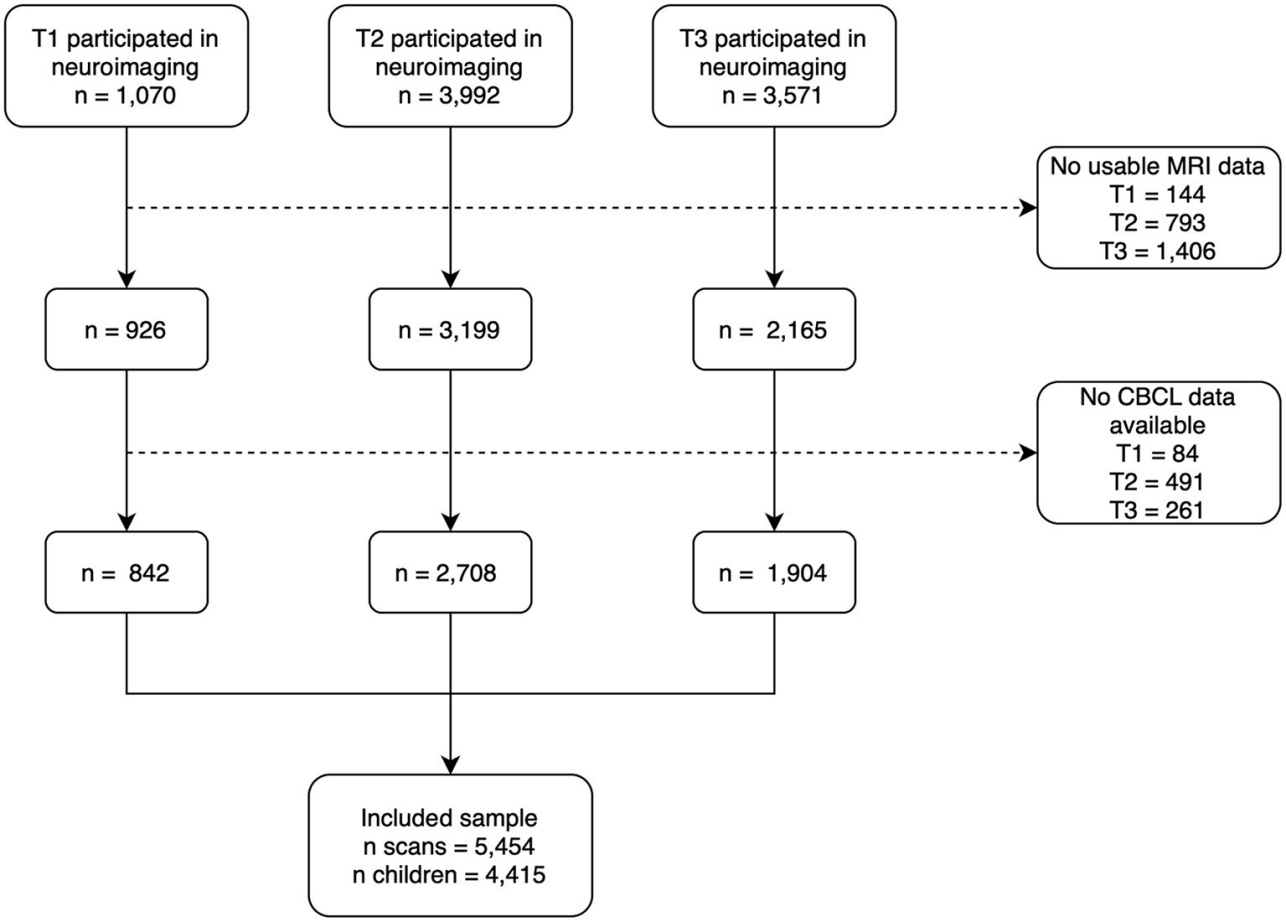
Flowchart of the study sample.

This study is embedded in the neuroimaging component of the Generation R Study, a large, longitudinal, population-based cohort with an observational design. The recruitment strategy has been described elsewhere (32–34). In brief, women living within specific zip codes of Rotterdam with a delivery date between April 2002 and January 2006 were invited to participate. The families are still being followed. When children were 6–10 years old (T1), 8–12 years old (T2), and 13 to 16 years old (T3), neuroimaging and behavioral data were collected. Children were included in the current study if they had good quality neuroimaging and behavioral data available in at least one wave of data collection. Neuroimaging data were excluded if any of the following conditions were present: dental braces, incidental findings that significantly alter brain morphology, or poor image quality. At T1, a total of 842 children were included, at T2, 2,708 children were included and at T3, 1,904 were included, resulting in a total sample of 5,454 scans from 4,415 children. A flowchart of the study sample is provided in Figure 1. The Generation R Study was approved by the medical ethics committee at the Erasmus MC and conducted according to the Declaration of Helsinki. Written informed consent, and when applicable assent, was obtained from the caregivers and their children.

### 2.2. Measures

#### 2.2.1. Behavioral Assessment

Child behavior was assessed using the Child Behavior Checklist (CBCL). At T1, the CBCL version for children aged 1.5–5 years was used (35), and at T2 and T3, the CBCL version for children aged 6–18 years was used (36). Both versions are reliable and valid questionnaires to assess child behavior (35, 36). The CBCL v1.5-5 has 99 items, and v6-18 has 112 items that are scored on a three-point Likert scale (0 = not true, 1 = somewhat true, 2 = very true). From the CBCL v1.5-5, seven empirically derived syndrome scales were calculated. From the CBCL v6-18, eight syndrome scales were obtained. These syndrome scales were summed into three broad domains of psychopathology [internalizing, externalizing and dysregulation profile (DP) symptoms]. In the CBCL v1.5-5, the internalizing scale includes the emotionally reactive, anxious/depressed, withdrawn and somatic complaints syndrome scales, and in the CBCL v6-18 it is a sum-score of the anxious/depressed, withdrawn/depressed and somatic complaints syndrome scales. Externalizing symptoms were assessed with the attention problems and aggressive behavior syndrome scales in CBCL v1.5-5, and with the rule-breaking behavior and aggressive behavior syndrome scales in v6-18. Lastly, the DP is a comorbid profile, which is the summed score of the anxious/depressed, attention problems and aggressive behavior syndrome scales in both CBCL versions (35, 36).

#### 2.2.2. MRI Acquisition

Neuroimaging data were collected on two scanners. At T1, structural MRI scans were acquired on a 3.0 Tesla GE Discovery MR750 MRI System (General Electric, Milwaukee, WI, USA). At T2 and T3, structural MRI scans were collected using a 3.0 Tesla GE Discovery MR750w MRI System (General Electric, Milwaukee, WI, USA). In all waves, we used an 8-channel receive only head coil. At T1, images were acquired using an inversion recovery fast spoiled gradient recalled (IR-FSPGR) sequence (sequence parameters: TE: 4.2 ms, TR: 10.3 ms, TI: 350 ms, flip angle: 16°, acquisition time: 5 min 40 s, FOV: 230.4 x 230.4, in-plane resolution: 0.9 mm^3^, coverage: whole-brain) (33). At T2 and T3, images were acquired using a 3D coronal inversion recovery fast spoiled gradient recalled (IR-FSPGR, BRAVO) sequence (sequence parameters: TE: 3.4 ms, TR: 8.77 ms, TI: 600 ms, flip angle: 10°, acquisition time: 5 min 20 s, FOV: 220 x 220, in-plane resolution: 1.0 mm^3^, phase encoding: R/L, fat suppression: yes, coverage: whole-brain) (34).

#### 2.2.3. MRI Processing

Image processing for data from T1 to T3 was performed using FreeSurfer analysis suite v6.0.0 (http://surfer.nmr.mgh.harvard.edu/). All images were processed individually using FreeSurfer. FreeSurfer processing steps have been described in detail previously (37). Briefly, the analysis stream includes converting raw DICOM data to “MGZ-files,” skull stripping, intensity normalization, and voxel segmentation of gray matter, white matter and cerebrospinal fluid. Labeling of the gray matter regions was performed using the Desikan-Killiany atlas (38).

#### 2.2.4. MRI Quality Assurance

MRI quality assurance has been described previously (33, 34, 39). To summarize, FreeSurfer image reconstructions were visually inspected by at least one rater. Based on how well FreeSurfer delineated the gray-white matter and the outer gray matter boundaries, each scan was rated on a Likert scale. Raters included master students, PhD students and postdoctoral researchers, who were all trained extensively, which was completed after correctly rating 30 scans of which quality was determined previously. At T1 and T2, scans were rated on a five point Likert scale (unusable, poor, sufficient, good, excellent). At T3, scans were rated on a three point Likert scale (poor, questionable, good). All scans that were unusable or of poor quality were excluded from the analyses. Quality assessment based on visual inspection was also compared to an automated quality assessment, which has been described previously for T1 and T2 data (40). Visual ratings were also compared to this automated quality assessment at T3, as well as to the Euler number which can be extracted after FreeSurfer reconstruction (41), this comparison is depicted in Supplementary Figure S1.

#### 2.2.5. Covariates

Multiple covariates were included in the analyses. Sex was derived from medical records at birth. Handedness was measured at each data collection wave, with the Edinburgh Handedness Inventory (EHI) (42), from which a laterality quotient was obtained ranging from –1 (fully left-handed) to +1 (fully right-handed). Maternal education, household income and child national origin were assessed using a questionnaire. Maternal education and household income were assessed at T1 and used as proxies for socioeconomic status (SES). Maternal education was divided into three categories: low (no education/primary school), middle (high school/vocational training), and high (higher vocational training/university) and household income into two categories: below €2000,- per month and above €2000,- per month. Child national origin was assessed at baseline, based on the birth country of the parents, it was categorized as Dutch and non-Dutch (African, American western, American non-western, Asian western, Asian non-western, Cape Verdean, Dutch Antilles, European, Indonesian, Moroccan, Oceania, Surinamese and Turkish).

### 2.3. Statistical Analyses

Our primary analyses assessed the relationship between deviations from typical development in cortical and subcortical regions of interest (ROIs) and multiple domains of psychopathology, using normative modeling. Specifically we included the following ROIs: the ACC (sum of rostral and caudal ACC), OFC (sum of lateral and medial OFC), rostral middle frontal cortex, the amygdala, hippocampus and the striatum (sum of putamen, caudate and nucleus accumbens). For cortical ROIs, we included measures of cortical thickness as well as cortical gray matter volume, for subcortical ROIs, gray matter volumes were included. In our secondary analyses, we explored the remaining (sub-)cortical regions labeled within FreeSurfer (38, 43), following the same procedure as for our primary analyses. To reduce the total number of tests, brain measures were averaged across both hemispheres.

The analyses consisted of five steps. First, we residualized brain morphology measures for possible covariate effects using two different models. In model 1 the effects of sex and handedness were regressed out of brain and CBCL measures, in model 2 the effects of SES and child national origin were additionally regressed out. Second, these residualized brain morphology measures were used to fit our normative model. A common way to fit a normative model is to use Gaussian process regression with age, and have the model predict the brain measure from those inputs. Generally, the subjects used in these analyses are considered to have typical development and the model is then validated using a held out subset of typically developing subjects (14–16). The Generation R study, however, is a population-based sample that is not enriched for children with psychopathology. Thus, we fit the model on all participants, which has been described as a viable option previously (15). Importantly, the current sample did not merely include cross-sectional data, but also longitudinal data for many of the participants. We leveraged the longitudinal data by bootstrapping multiple unique combinations of subsets of scans as training sets. In the individual training sets, all participants were only included once, to prevent overfitting on a single person's development. To reach an approximately even distribution of participants across ages in each training set, approximately 50% of the scans acquired at T2 and T3 were not included in each individual training set. In each test set, 10% of the participants from T1, T2, and T3 were included.

We used Gaussian process regression (GPR) to fit (non)-linear normative trajectories to each brain measure across age. GPRs fit a Gaussian process to the given data points, such that any age, along a continuum (x-axis), is associated with a normal distribution for each brain region (y-axis). This approach is especially well-suited for normally distributed data. Given that we used a population sample, we assume that points for each brain region are normally distributed. Each brain measure was thus fit using a separate Gaussian process with GPytorch's (44) exact Gaussian processes module. The Gaussian process is continuous and can interpolate and extrapolate from the age range of a given set of points. An added benefit of associating a normal distribution with each brain measure given a certain age is that we can use the standard deviation of the distribution to calculate how confident the Gaussian process is in its prediction. It also allows us to interpret the distributions over time as each brain measure's normative trajectory. An important hyperparameter for Gaussian process regression is its kernel, which determines the shape of the line that the normal distributions are centered on. We empirically evaluate a variety of typical kernels for each brain region to limit assumptions about their normative trajectory. Before fitting the GPR, all brain measures were standardized to a mean of 0 and a standard deviation of 1, to accommodate direct comparisons of effect-size estimates across brain regions and measures. The age was rescaled between 0 and 1 based on the minimum and maximum age in the dataset. To assure we obtain the best fit for the trajectory of a given region, we evaluated multiple types of kernels on an unseen validation set. This validation set was a small (10% of each wave in the training fold) subsample of the training set. The kernels we used included a linear, Matern, radial basis function (RBF), and a rational quadratic kernel. We averaged the performance of each kernel over the validation sets to select the best kernel. The best kernel and complete training set, including the validation set, were used to train the final model. The final models for each training set were then used to predict the mean and standard deviance at each age in the test fold.

Third, the difference between these predicted mean and standard deviations for each morphological value and the true morphological values were used to calculate the z-scores for each participant. The formula for this calculation is shown in Equation 1.


(1) $$z=\frac{y-\hat{y}}{\hat{\sigma }}$$

Where, **y** are the true values for the brain measures at each age, $\hat{y}$ are the mean predicted brain measures at each age, and $\hat{\sigma }$ are the predicted variances at each age. Note that because the normative model predicts a normal distribution at each age, average predicted brain measure at each age is the most likely value of that brain measure. Fourth, the association between the deviations of each individual from normative development and psychopathology was tested, using separate linear mixed model analyses. Internalizing, externalizing and DP symptoms were entered as dependent variables, z-scores for all brain measures were entered as independent variables, and a random effect was applied for participant ID. Finally, these analyses were repeated with an interaction term for age, to assess whether differences in the slope of deviations from typical development were age-dependent.

To assess the robustness of the findings, two sensitivity analyses were performed. First, normative development curves for (sub-)cortical volume in each region were fit with the effect of total intracranial volume (ICV) regressed out of individual volumes. Z-scores obtained from this model were subsequently related to psychopathology symptoms, to assess whether the effects observed were global or specific to regions. Second, we assessed whether deviations from typical development are specific to psychopathology domains. Therefore, analyses were repeated for brain regions that showed a significant relationship with two or more individual psychopathology domains, in which all significant psychopathology domains were entered in the model simultaneously.

Lastly, as *post-hoc* analyses, both normative developmental trajectories and deviations due to psychopathology were established for surface area in all hypothesis-driven and exploratory regions of interest, after which deviations from typical development were related to all psychopathology domains.

Bootstrapping and regression analyses were performed in R version 3.6.3 (45), normative modeling was performed in Python version 3.9.0 (46). Missing data in the covariates were imputed 30 times with 30 iterations using multiple imputation through chained equations with the mice package (47). The false discovery rate (FDR) was controlled using the Benjamini Hochberg procedure (48). Primary analyses were corrected for a total of 27 tests, at q-value = 0.05. Exploratory analyses were separately corrected for a total of 180 tests (q-value = 0.05). Analyses using an interaction term were corrected for multiple testing following the same procedure for a total of 207 tests. Hypotheses and analyses for this project were publicly preregistered, a time-stamped version of this preregistration is available via: www.osf.io/aqc4s. Slight deviations from our initial preregistration are described in the Supplementary Material. Analysis scripts are publicly available via https://github.com/eloygeenjaar/normative-smri-psychopathology.

## 3. Results

### 3.1. Sample Characteristics

Sample characteristics are described in Table 1. At all time points the majority of the children included were of Dutch national origin (T1 = 70.8%, T2 = 65.4%, T3 = 63.0%), had mothers with high educational levels (T1 = 56.7%, T2 = 61.9%, T3 = 59.8%) and came from households with an income >€2,000.- per month (T1 = 76.7%, T2 = 80.5%, T3 = 78.8%).

**Table 1:** Demographic characteristics.

	**T1**		**T2**		**T3**	
	***n***		***n***		***n***	
Age MRI (Mean, SD)	842	7.96 (1)	2,708	10.1 (0.57)	1,904	14 (0.6)
Age CBCL (Mean, SD)	842	6.06 (0.45)	2,708	9.7 (0.28)	1,904	13.52 (0.36)
**Measures in %**						
*Child national origin*						
Dutch	596	70.78%	1,771	65.4%	1,199	62.97%
Non Dutch	246	29.22%	937	34.6%	705	37.03%
*Maternal education*						
Low	30	3.56%	47	1.74%	57	2.99%
Middle	335	39.79%	986	36.41%	708	37.18%
High	477	56.65%	1675	61.85%	1139	59.82%
Household income						
< €2,000.- per month	196	23.28%	528	19.5%	404	21.22%
>€2,000.- per month	646	76.72%	2,180	80.5%	1,500	78.78%
**Measures in median, IQR**						
Handedness	842	0.82 (0.64–0.92)	2708	0.83 (0.67–1)	1,904	0.83 (0.67–1)
*Child psychopathology*						
Internalizing	842	6 (2–11)	2,708	3 (1–7)	1,904	4 (1–8)
Externalizing	842	8 (3–15)	2,708	2 (0–5)	1,904	2 (0–6)
Dysregulation Profile	842	10 (4–18)	2,708	6 (3–11)	1,904	6 (3-12)

### 3.2. Normative Development of Brain Morphology

**Figure 2:**
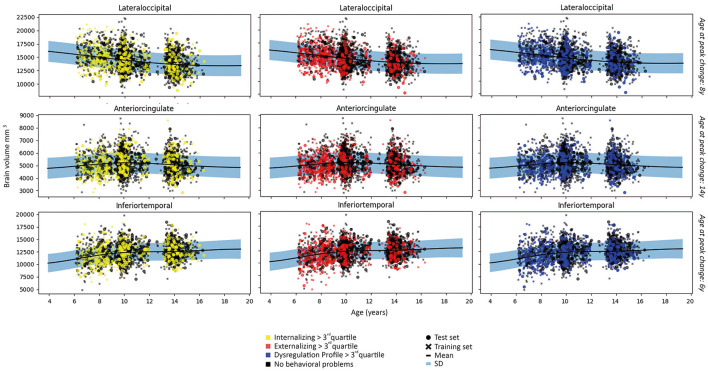
Examples of patterns of (sub-)cortical volume for each subject in the first fold and the fit of the normative model to the (sub-)cortical volume in the training set. A wider prediction range outside of data distribution is used for visualization purposes. For illustrative purposes, measures are rescaled to the original means and standard deviations.

**Figure 3:**
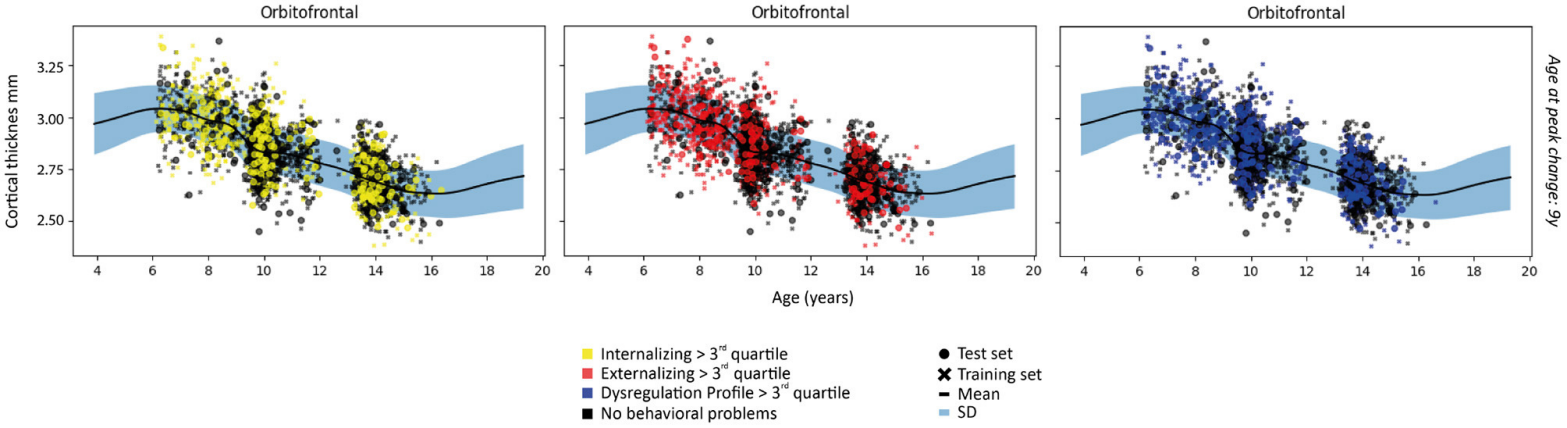
Examples of patterns of cortical thickness for each subject in the first fold and the fit of the normative model to the cortical thickness in the training set. A wider prediction range outside of data distribution is used for visualization purposes. For illustrative purposes, measures are rescaled to the original means and standard deviations.

**Figure 4:**
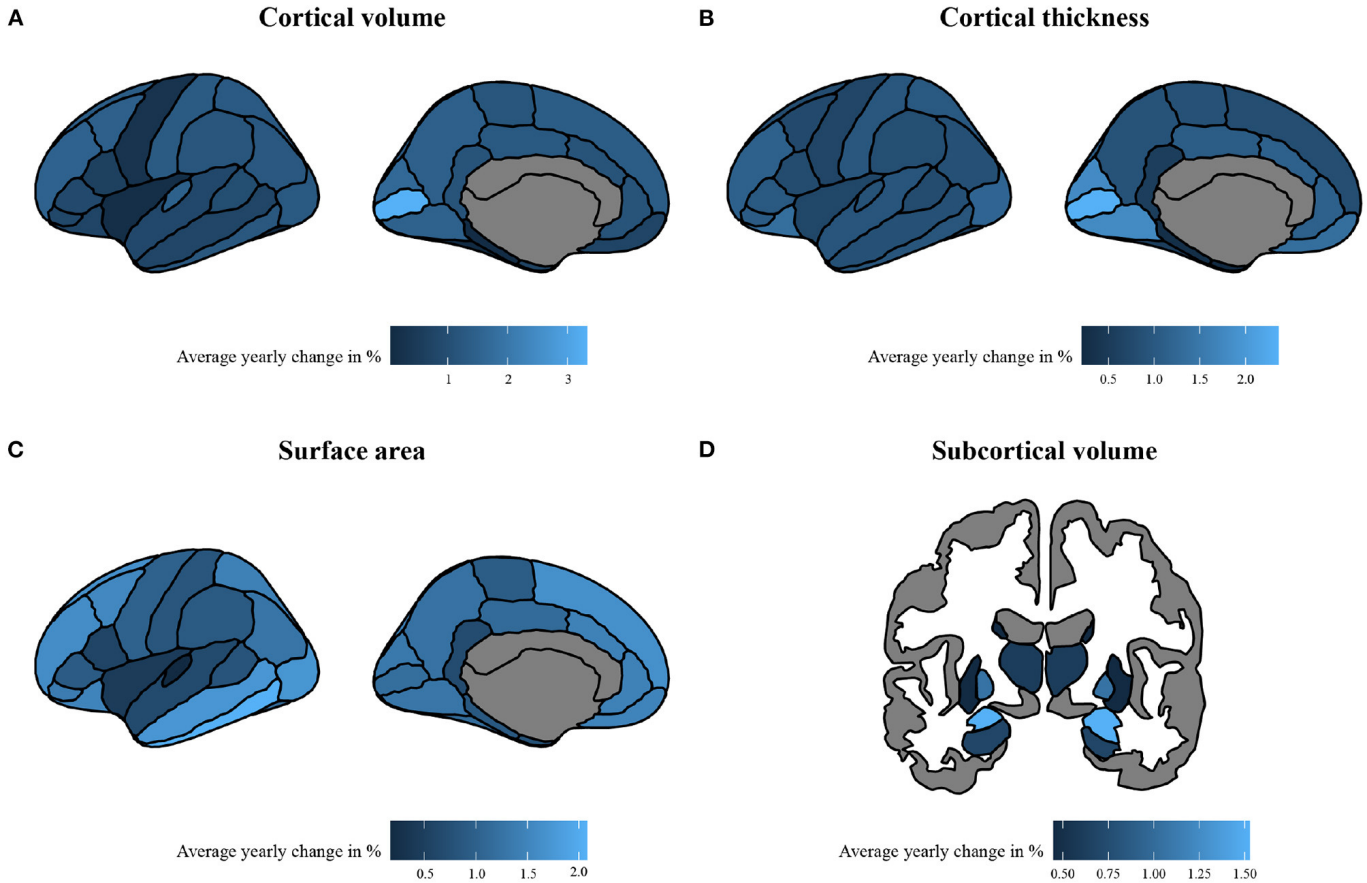
The absolute average percentage change per year of morphological measures. The change is calculated with absolute percentages of change per year, calculated across a span of 6–16 years in **(A)** cortical volume, **(B)** cortical thickness, **(C)** surface area, and **(D)** subcortical volume. To enhance interpretability, absolute average change was calculated using brain measures that were rescaled to their original means and standard deviations.

The linear and/or non-linear development curves were fit for each residualized region (model 1 and model 2) included in the Desikan-Killiany atlas (38). Examples of the most common patterns that we observed are depicted in Figures 2, 3. Full results from the normative model are shown in Supplementary Figures S2, S3. The average change in (sub-)cortical volume and cortical thickness across 6–16 years of age is provided in Figure 4.

#### 3.2.1. Cortical and Subcortical Volume

Normative development curves between age 6–16 revealed an increasing slope for (sub-)cortical volume in the entorhinal, inferior temporal, middle temporal, temporal pole, hippocampus, pallidum and thalamic regions; a decreasing slope for the cuneus, frontal pole, isthmus cingulate, lateral occipital, lingual, paracentral, pars opercularis, pars triangularis, pericalcarine, precuneus, post central, supramarginal and transverse temporal regions; and an inverted U-shaped curve for the anterior cingulate, banks of superior temporal sulcus, caudal middle frontal, inferior parietal, orbitofrontal, pars orbitalis, precentral, posterior cingulate, rostral middle frontal, superior frontal, superior parietal, superior temporal, amygdala and striatal regions. Flat trajectories were observed in the fusiform, insula and parahippocampal regions. These patterns were consistent across model 1 and 2. Given that normative development curves were fit on 12 bootstrapped folds of the dataset, the optimal fit differed slightly between individual folds, however, patterns described were consistent across all folds.

#### 3.2.2. Cortical Thickness

The normative models fit on cortical thickness data showed a decreasing slope from early to later neurodevelopment in the majority of regions (see Supplementary Figure S1). The steepest slope was primarily seen between 6 and 12 years of age. Noteworthy exceptions with a fairly flat slope across neurodevelopment were the entorhinal and temporal pole regions. These patterns were consistent across models and folds.

### 3.3. Deviations From Normative Development and Psychopathology

#### 3.3.1. Hypothesis Driven Analyses

##### 3.3.1.1. Cortical and Subcortical Volume

**Figure 5:**
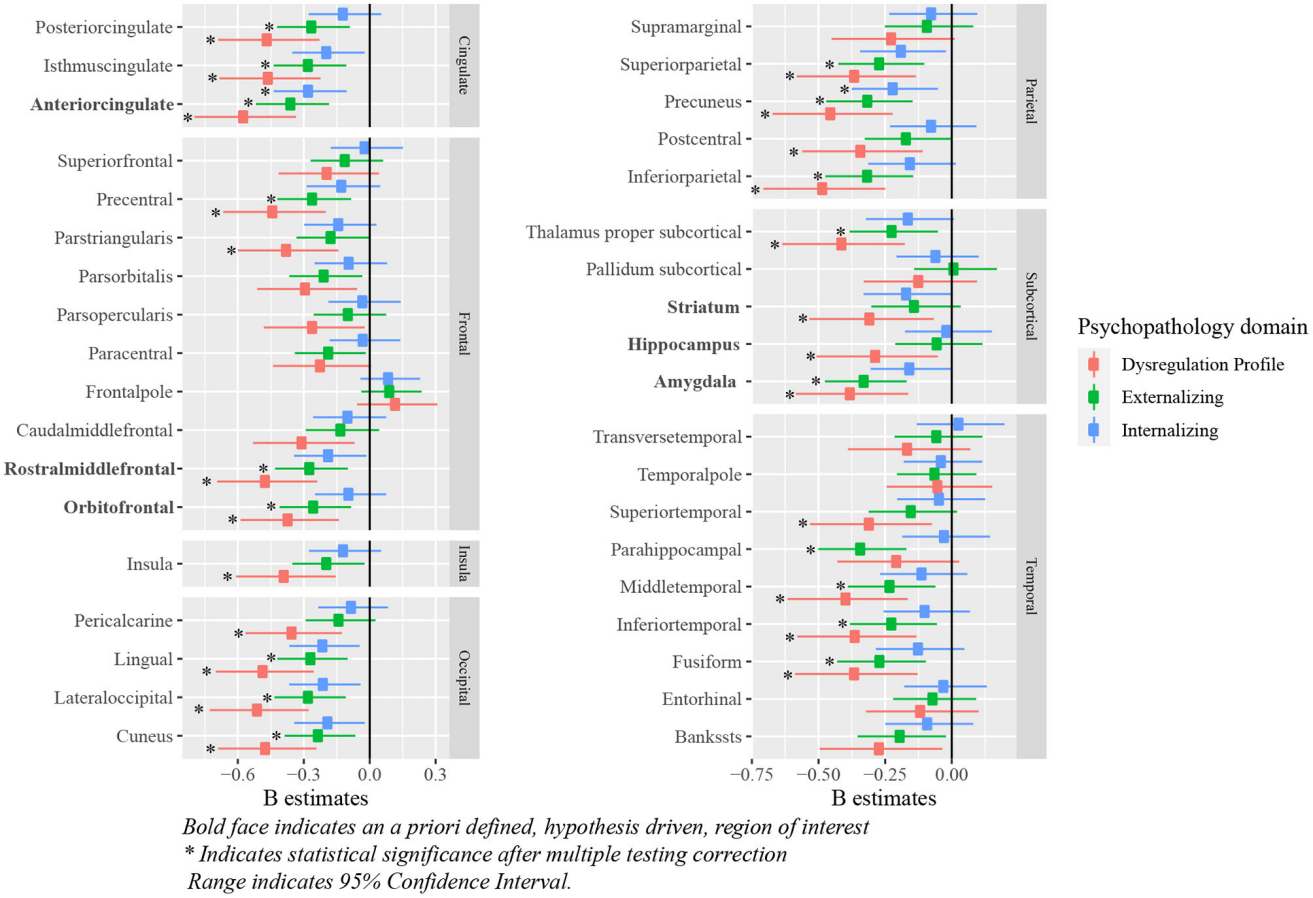
Standardized B estimates of the relationship between z-scores (representing deviations of typical development in (sub-)cortical volume) and psychopathology symptoms. Bold face indicates an a priori defined, hypothesis driven, region of interest *indicates statistical significance after multiple testing correction range indicates 95% confidence interval.

All a priori selected, hypothesis driven, regions of interest for cortical and subcortical volume showed a negative relationship with some psychopathology domains, meaning that psychopathology symptoms were related to negative deviations from typical brain development. After correction for multiple testing, negative deviations from typical development in the ACC were related to all psychopathology domains; negative deviations in the OFC, the rostral middle frontal cortex, and the amygdala were related to externalizing and DP symptoms. Lastly, negative deviations from typical development in hippocampal and striatal volume were related to DP symptoms. Full results are shown in Figure 5 (model 2), Table 2 and Supplementary Table S1 (model 1).

**Table 2:** Hypothesis driven (sub-)cortical volume model 2.

**Brain region**	**Psychopathology symptoms**	**B**	**S.E**.	***p*** **-value**	
Anterior cingulate	Internalizing	–0.271	0.085	1.39e-03*
	Externalizing	–0.351	0.085	3.61e-05*
	Dysregulation Profile	–0.566	0.118	1.71e-06*
Orbitofrontal	Internalizing	–0.087	0.083	2.92e-01
	Externalizing	-0.248	0.083	2.91e-03*
	Dysregulation Profile	–0.364	0.114	1.42e-03*
Rostral middle frontal	Internalizing	–0.18	0.084	3.28e-02
	Externalizing	–0.265	0.084	1.73e-03*
	Dysregulation Profile	–0.467	0.116	6.03e-05*
Amygdala	Internalizing	–0.152	0.078	5.19e-02
	Externalizing	–0.323	0.078	3.92e-05*
	Dysregulation Profile	–0.374	0.107	5.05e-04*
Hippocampus	Internalizing	–0.012	0.083	8.81e-01
	Externalizing	–0.048	0.084	5.62e-01
	Dysregulation Profile	–0.28	0.116	1.6e-02*
Striatum	Internalizing	–0.163	0.085	5.57e-02
	Externalizing	–0.134	0.086	1.18e-01
	Dysregulation Profile	–0.301	0.119	1.16e-02*

* *Indicates significance after correction for multiple testing using FDR-BH at a q-value of 0.05*.

##### 3.3.1.2. Cortical Thickness

**Figure 6:**
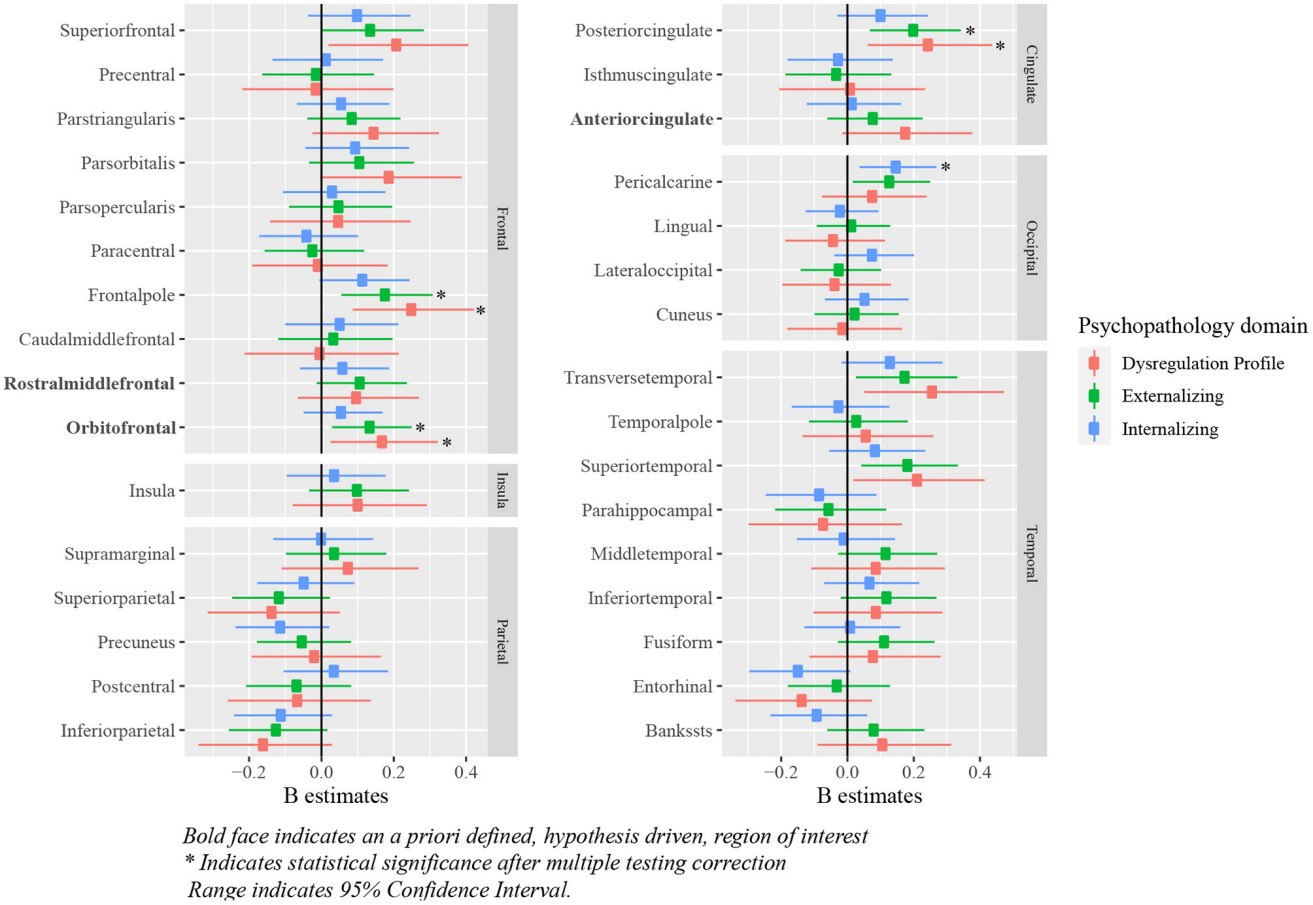
Standardized B estimates of the relationship between z-scores (representing deviations of typical development in cortical thickness) and psychopathology symptoms. Bold face indicates an a priori defined, hypothesis driven, region of interest *indicates statistical significance after multiple testing correction range indicates 95% confidence interval.

Positive associations were observed between deviations from typical cortical thickness development in the OFC, and externalizing and DP symptoms after correction for multiple testing. No associations were observed between deviations from normative development in the ACC and rostral middle frontal cortex, and each of the psychopathology domains. Full results are shown in Figure 6 (model 2), Table 3 and Supplementary Table S2 (model 1).

**Table 3:** Hypothesis driven cortical thickness model 2.

**Brain region**	**Psychopathology symptoms**	**B**	**S.E**.	***p*** **-value**
Anterior cingulate	Internalizing	0.02	0.073	7.86e-01
	Externalizing	0.083	0.073	2.56e-01
	Dysregulation Profile	0.181	0.1	7.11e-02
Orbitofrontal	Internalizing	0.06	0.056	2.83e-01
	Externalizing	0.139	0.056	1.33e-02*
	Dysregulation Profile	0.173	0.076	2.22e-02*
Rostral middle frontal	Internalizing	0.064	0.063	3.11e-01
	Externalizing	0.112	0.064	7.89e-02
	Dysregulation Profile	0.102	0.086	2.34e-01

* *Indicates significance after correction for multiple testing using FDR-BH at a q-value of 0.05*.

#### 3.3.2. Exploratory Analyses

##### 3.3.2.1. Cortical and Subcortical Volume

Exploratory analyses revealed significant negative associations between deviations from typical development in several (sub-)cortical volume regions and psychopathology domains. After correction for multiple testing, all psychopathology domains were related to negative deviations from typical development in the precuneus. Negative deviations from typical development in the cuneus, fusiform, inferior parietal, inferior temporal, isthmus cingulate, lateral occipital, lingual, middle temporal, posterior cingulate, precentral, superior parietal and thalamus were observed for externalizing and DP symptoms. Lastly, negative deviations in the parahippocampal region were specific to externalizing symptoms and negative deviations in the insula, pars triangularis, pericalcarine, postcentral and superior temporal region were specific to DP symptoms. Full results are shown in Figure 5 (model 2), Table 4 and Supplementary Table S3 (model 1).

**Table 4:** Exploratory (sub-)cortical volume model 2.

	**Internalizing**			**Externalizing**			**Dysregulation profile**		
**Brain region**	**B**	**S.E**.	***p*** **-value**	**B**	**S.E**.	***p*** **-value**	**B**	**S.E**.	***p*** **-value**
Banks of the superior temporal sulcus	–0.084	0.084	3.19e-01	–0.188	0.085	2.7e-02	–0.266	0.117	2.37e-02
Caudal middle frontal	–0.092	0.085	2.8e-01	–0.124	0.085	1.45e-01	−0.3	0.118	1.09e-02
Cuneus	–0.183	0.082	2.58e-02	–0.226	0.082	5.96e-03*	-0.466	0.114	4.63e-05*
Entorhinal	–0.024	0.079	7.65e-01	–0.064	0.08	4.2e-01	–0.111	0.108	3.06e-01
Frontal pole	0.093	0.069	1.77e-01	0.099	0.07	1.58e-01	0.125	0.093	1.8e-01
Fusiform	–0.119	0.085	1.61e-01	–0.263	0.085	1.99e-03*	-0.358	0.117	2.28e-03*
Inferior parietal	–0.149	0.084	7.57e-02	–0.31	0.084	2.39e-04*	–0.478	0.117	4.35e-05*
Inferior temporal	–0.094	0.083	2.57e-01	–0.219	0.083	8.56e-03*	–0.356	0.114	1.85e-0*
Insula	–0.112	0.084	1.81e-01	–0.188	0.084	2.55e-02	-0.381	0.116	9.99e-04*
Isthmus cingulate	–0.188	0.084	2.52e-02	–0.272	0.084	1.27e-03*	–0.454	0.117	1.15e-04*
Lateral occipital	–0.204	0.083	1.38e-02	–0.271	0.083	1.08e-03*	–0.503	0.115	1.3e-05*
Lingual	–0.206	0.081	1.17e-02	–0.26	0.082	1.46e-03*	–0.478	0.114	2.74e-05*
Middle temporal	–0.105	0.084	2.08e-01	–0.226	0.084	7.23e-03*	–0.391	0.115	6.96e-04*
Paracentral	–0.022	0.082	7.9e-01	–0.179	0.082	2.96e-02	–0.217	0.114	5.74e-02
Parahippocampal	–0.021	0.084	8.02e-01	–0.336	0.084	7.29e-05*	–0.201	0.117	8.56e-02
Pars opercularis	–0.024	0.084	7.77e-01	–0.09	0.084	2.86e-01	–0.253	0.117	3.11e-02
Pars orbitalis	–0.086	0.084	3.07e-01	–0.2	0.085	1.84e-02	-0.285	0.116	1.42e-02
Pars triangularis	-0.134	0.084	1.11e-01	-0.168	0.084	4.51e-02	–0.371	0.116	1.47e-03*
Pericalcarine	–0.076	0.081	3.48e-01	–0.132	0.081	1.03e-01	–0.346	0.112	2.02e-03*
Posterior cingulate	–0.113	0.084	1.81e-01	–0.256	0.085	2.48e-03*	–0.459	0.118	1.01e-04*
Postcentral	–0.07	0.083	4e-01	–0.164	0.083	4.79e-02	–0.335	0.115	3.67e-03*
Precentral	–0.12	0.085	1.62e-01	–0.253	0.086	3.24e-03*	–0.433	0.119	2.71e-04*
Precuneus	–0.214	0.082	9.7e-03*	–0.309	0.083	1.92e-04*	–0.448	0.115	1.02e-04*
Superior frontal	–0.013	0.084	8.76e-01	–0.104	0.084	2.17e-01	–0.186	0.117	1.1e-01
Superior parietal	–0.183	0.082	2.61e-02	–0.265	0.082	1.36e-03*	–0.358	0.114	1.69e-03*
Superior temporal	–0.04	0.084	6.35e-01	–0.146	0.085	8.47e-02	–0.303	0.117	9.43e-03*
Supramarginal	–0.069	0.084	4.11e-01	–0.085	0.085	3.14e-01	–0.22	0.118	6.2e-02
Temporal pole	–0.033	0.075	6.62e-01	–0.057	0.076	4.56e-01	–0.046	0.101	6.49e-01
Transverse temporal	0.033	0.084	6.91e-01	–0.05	0.084	5.54e-01	–0.16	0.117	1.73e-01
Pallidum	–0.053	0.079	5e-01	0.014	0.079	8.55e-01	–0.118	0.108	2.77e-01
Thalamus	–0.157	0.084	6.31e-02	–0.218	0.085	1.01e-02*	–0.406	0.117	5.43e-04*

* *Indicates significance after correction for multiple testing using FDR-BH at a q-value of 0.05*.

##### 3.3.2.2. Cortical Thickness

Positive deviations were observed in the pericalcarine region in relation to internalizing symptoms. Additionally, positive deviations from typical development in the frontal pole and the posterior cingulate region were related to higher externalizing and DP symptoms. Deviations from typical development in cortical thickness for most regions were not related to psychopathology symptoms. Full results are shown in Figure 6 (model 2), Table 5 and Supplementary Table S4 (model 1).

**Table 5:** Exploratory cortical thickness model 2.

	**Internalizing**			**Externalizing**			**Dysregulation profile**		
**Brain region**	**B**	**S.E**.	***p*** **-value**	**B**	**S.E**.	***p*** **-value**	**B**	**S.E**.	***p*** **-value**
Banks of the superior temporal sulcus	–0.086	0.074	2.46e-01	0.086	0.075	2.51e-01	0.112	0.103	2.77e-01
Caudal middle frontal	0.056	0.08	4.81e-01	0.039	0.081	6.31e-01	0.001	0.109	9.95e-01
Cuneus	0.058	0.064	3.65e-01	0.028	0.065	6.66e-01	–0.009	0.088	9.2e-01
Entorhinal	–0.143	0.078	6.58e-02	–0.026	0.079	7.44e-01	–0.132	0.105	2.11e-01
Frontal pole	0.119	0.064	6.18e-02	0.182	0.064	4.83e-03*	0.254	0.086	2.99e-03*
Fusiform	0.015	0.074	8.43e-01	0.117	0.074	1.15e-01	0.083	0.101	4.09e-01
Inferior parietal	–0.106	0.069	1.24e-01	–0.12	0.07	8.54e-02	–0.156	0.094	9.9e-02
Inferior temporal	0.073	0.073	3.17e-01	0.124	0.074	9.22e-02	0.092	0.099	3.52e-01
Insula	0.041	0.07	5.58e-01	0.104	0.071	1.4e-01	0.106	0.095	2.63e-01
Isthmus cingulate	–0.022	0.081	7.88e-01	–0.028	0.081	7.36e-01	0.014	0.112	8.98e-01
Lateral occipital	0.081	0.061	1.9e-01	–0.02	0.062	7.49e-01	–0.032	0.084	6.99e-01
Lingual	–0.016	0.056	7.73e-01	0.018	0.056	7.48e-01	–0.037	0.077	6.29e-01
Middle temporal	–0.005	0.076	9.5e-01	0.121	0.076	1.11e-01	0.092	0.103	3.71e-01
Paracentral	–0.035	0.07	6.11e-01	–0.019	0.07	7.84e-01	–0.004	0.096	9.67e-01
Parahippocampal	–0.079	0.085	3.53e-01	–0.051	0.085	5.54e-01	–0.067	0.118	5.72e-01
Pars opercularis	0.035	0.072	6.26e-01	0.053	0.073	4.65e-01	0.052	0.099	5.99e-01
Pars orbitalis	0.099	0.073	1.77e-01	0.111	0.074	1.34e-01	0.192	0.1	5.48e-02
Pars triangularis	0.06	0.065	3.55e-01	0.09	0.066	1.72e-01	0.15	0.089	9.26e-02
Pericalcarine	0.152	0.059	1.01e-02*	0.133	0.06	2.6e-02	0.081	0.08	3.13e-01
Posterior cingulate	0.106	0.07	1.27e-01	0.205	0.07	3.48e-03*	0.249	0.096	9.56e-03*
Postcentral	0.04	0.074	5.86e-01	–0.063	0.074	3.97e-01	–0.061	0.101	5.45e-01
Precentral	0.018	0.078	8.17e-01	–0.009	0.079	9.07e-01	–0.01	0.107	9.27e-01
Precuneus	–0.108	0.066	1.02e-01	–0.048	0.066	4.68e-01	–0.014	0.091	8.76e-01
Superior frontal	0.104	0.072	1.48e-01	0.141	0.073	5.32e-02	0.213	0.099	3.13e-02
Superior parietal	–0.043	0.068	5.29e-01	–0.112	0.069	1.05e-01	–0.132	0.093	1.58e-01
Superior temporal	0.09	0.074	2.25e-01	0.187	0.074	1.18e-02	0.216	0.101	3.25e-02
Supramarginal	0.005	0.07	9.44e-01	0.041	0.071	5.64e-01	0.079	0.097	4.13e-01
Temporal pole	–0.02	0.075	7.86e-01	0.033	0.076	6.63e-01	0.062	0.101	5.4e-01
Transverse temporal	0.135	0.078	8.31e-02	0.179	0.078	2.21e-02	0.261	0.107	1.48e-02

* *Indicates significance after correction for multiple testing using FDR-BH at a q-value of 0.05*.

#### 3.3.3. Interaction Effect Age

After correction for multiple testing, a significant positive interaction effect between age and the deviations from typical development in cortical volume was observed in the fusiform and parahippocampal region in relation to externalizing symptoms. This indicates that with increasing age, deviations from typical development become larger in those with externalizing symptoms. Full results are shown in Supplementary Tables S5–S8.

#### 3.3.4. Sensitivity Analyses

In the first sensitivity analysis we repeated our analyses on cortical and subcortical volume while correcting for ICV, to assess whether the observed relationships were global or region specific. While the relationships attenuated in many regions, some region specific deviations were identified. In our hypothesis driven regions, the relationship between the ACC development and all psychopathology domains, as well as the relationship between the amygdala and externalizing symptoms remained statistically significant after adjustment for ICV. Additionally, exploratory analyses indicated a significant relationship between the lingual region and all psychopathology domains; between the cuneus, inferior parietal, lateral occipital and precuneus, and externalizing and DP symptoms; and between the isthmus cingulate, pars triangularis, pericalcarine and posterior cingulate, and DP symptoms. Full results are shown in Supplementary Tables S9, S10.

In the second sensitivity analysis we assessed whether, for those regions that showed a significant relationship with multiple psychopathology domains, certain psychopathology domains were associated to deviations from typical development above and beyond other psychopathology symptoms. Regarding cortical volume, our findings indicated that internalizing and externalizing symptoms are not related to deviations from typical development when controlling for other psychopathology domains. DP symptoms, however, were related to deviations from typical development in the ACC and the precuneus, when adjusting for internalizing and externalizing symptoms. Additionally, DP symptoms were related to deviations in the rostral middle frontal, cuneus, inferior parietal, isthmus cingulate, lateral occipital, middle temporal, posterior cingulate, precentral and thalamic region after adjustment for externalizing symptoms. For cortical thickness, none of the psychopathology symptoms were related when controlling for other psychopathology domains. Full results are shown in Supplementary Tables S11, S12.

#### 3.3.5. *Post-hoc* Analyses

**Figure 7:**
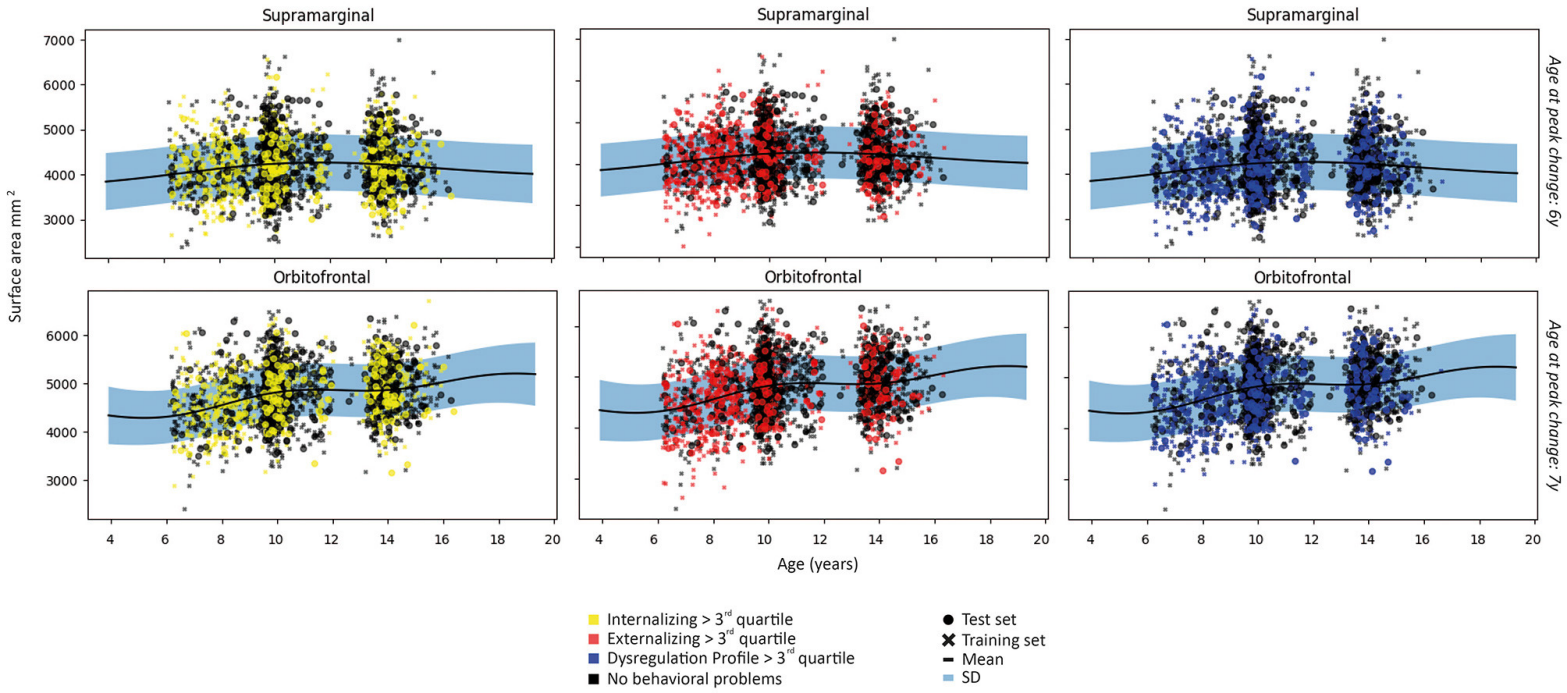
Examples of patterns of surface area for each subject in the first fold and the fit of the normative model to the surface area in the training set. A wider prediction range outside of data distribution is used for visualization purposes. For illustrative purposes, measures are rescaled to the original means and standard deviations.

As *post-hoc* analyses, normative trajectories were established for cortical surface area. Normative trajectories showed an inverted U-shaped relationship for the majority of regions. Notable exceptions were positive trajectories in the anterior cingulate, entorhinal, fusiform, inferior temporal, insula, middle temporal, orbitofrontal, parahippocampal, pars opercularis, pars orbitalis, pars triangularis and temporal pole; and a negative trajectory for the transverse temporal region. Examples of the most common patterns are shown in Figure 7 and full results from the normative model are shown in Supplementary Figure S4.

**Figure 8:**
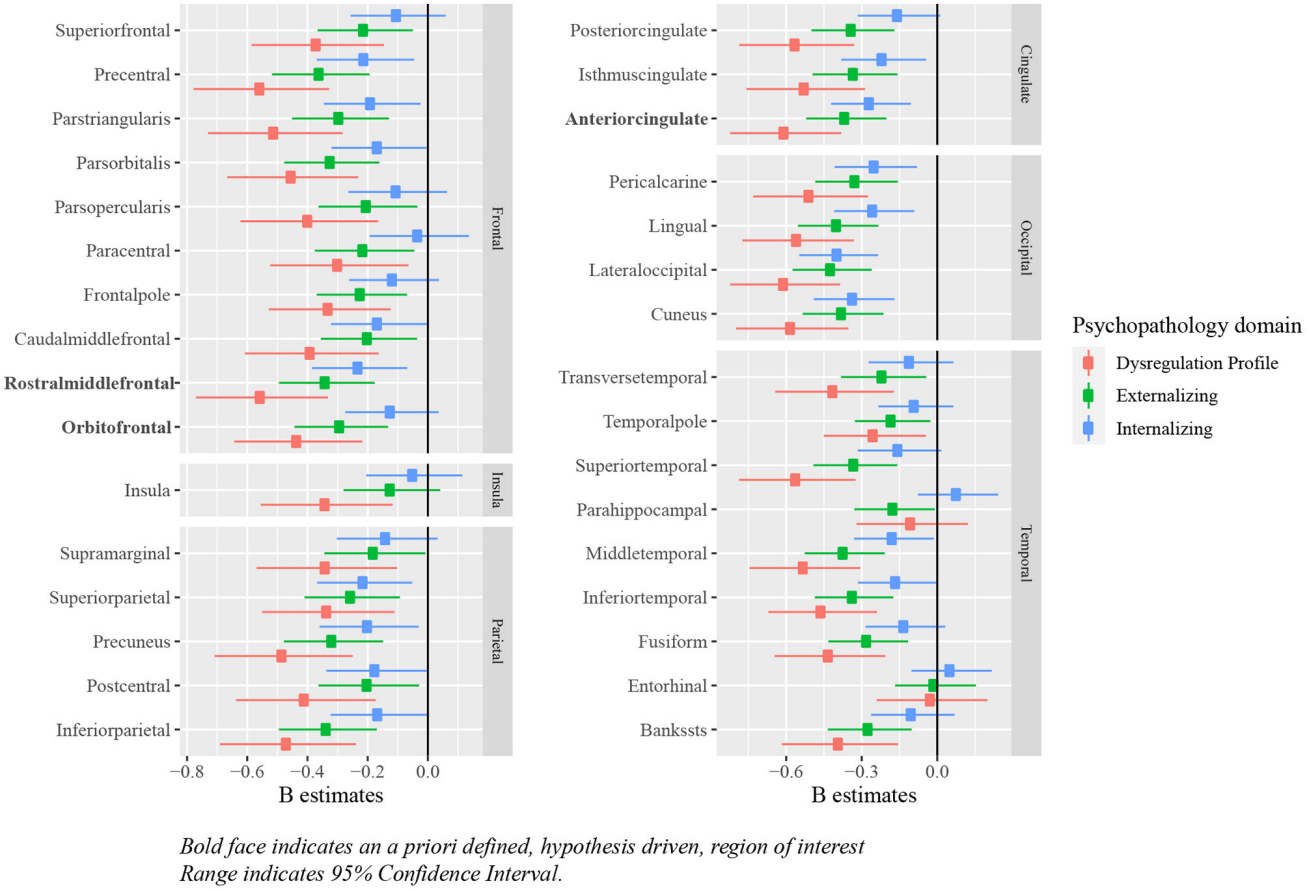
Standardized B estimates of the relationship between z-scores (representing deviations of typical development in surface area) and psychopathology symptoms. Bold face indicates an a priori defined, hypothesis driven, region of interest range indicates 95% confidence interval.

Relationships between deviations from typical development of surface area and psychopathology largely mirrored the associations between cortical volume and psychopathology, in which most regions showed negative deviations from typical development. Full results are presented in Figure 8 and Tables 6, 7.

**Table 6:** Hypothesis driven surface area model 2.

**Brain region**	**Psychopathology symptoms**	**B**	**S.E**.	***p*** **-value**
Anterior cingulate	Internalizing	–0.263	0.081	1.17e-03
	Externalizing	–0.361	0.081	9.13e-06
	Dysregulation Profile	–0.602	0.113	1e-07
Orbitofrontal	Internalizing	–0.119	0.079	1.31e-01
	Externalizing	–0.288	0.079	3e-04
	Dysregulation Profile	–0.43	0.109	7.68e-05
Rostral middle frontal	Internalizing	–0.227	0.081	5.09e-03
	Externalizing	–0.336	0.081	3.62e-05
	Dysregulation Profile	–0.551	0.112	9.35e-07

**Table 7:** Exploratory surface area model 2.

**Brain region**	**B**	**S.E**.	***p*** **-value**	**B**	**S.E**.	***p*** **-value**	**B**	**S.E**.	***p*** **-value**
Banks of the superior temporal sulcus	–0.097	0.085	2.52e-01	–0.268	0.085	1.6e-03	–0.386	0.118	1.06e-03
Caudal middle frontal	–0.162	0.081	4.63e-02	–0.196	0.082	1.66e-02	–0.386	0.113	6.88e-04
Cuneus	–0.33	0.082	5.72e-05	–0.374	0.082	5.63e-06	–0.576	0.114	4.77e-07
Entorhinal	0.057	0.081	4.84e-01	–0.007	0.082	9.3e-01	–0.021	0.112	8.53e-01
Frontal pole	–0.112	0.076	1.4e-01	–0.219	0.077	4.26e-03	–0.326	0.103	1.64e-03
Fusiform	–0.127	0.081	1.16e-01	–0.274	0.081	7.17e-04	–0.426	0.112	1.5e-04
Inferior parietal	–0.161	0.083	5.2e-02	–0.332	0.083	6.5e-05	–0.465	0.115	5.86e-05
Inferior temporal	–0.159	0.079	4.5e-02	–0.33	0.08	3.45e-05	–0.455	0.11	3.62e-05
Insula	–0.045	0.082	5.81e-01	–0.12	0.082	1.45e-01	–0.336	0.112	2.68e-03
Isthmus cingulate	–0.213	0.086	1.35e-02	–0.327	0.086	1.54e-04	–0.522	0.12	1.47e-05
Lateral occipital	–0.391	0.08	1.06e-06	–0.417	0.08	2.15e-07	–0.604	0.111	6.54e-08
Lingual	–0.25	0.081	2.09e-03	–0.394	0.081	1.42e-06	–0.552	0.113	1.11e-06
Middle temporal	–0.172	0.081	3.32e-02	–0.367	0.081	6.35e-06	–0.526	0.112	3.05e-06
Paracentral	–0.028	0.084	7.36e-01	–0.211	0.084	1.27e-02	–0.294	0.117	1.22e-02
Parahippocampal	0.082	0.081	3.11e-01	–0.17	0.082	3.75e-02	–0.1	0.113	3.77e-01
Pars opercularis	–0.1	0.084	2.31e-01	–0.199	0.084	1.78e-02	–0.394	0.117	7.65e-04
Pars orbitalis	–0.162	0.08	4.36e-02	–0.319	0.081	8.05e-05	–0.449	0.111	5.65e-05
Pars triangularis	–0.185	0.082	2.41e-02	–0.29	0.082	4.24e-04	–0.507	0.114	9.36e-06
Pericalcarine	–0.244	0.083	3.5e-03	–0.321	0.084	1.33e-04	–0.503	0.116	1.59e-05
Posterior cingulate	–0.152	0.084	6.98e-02	–0.335	0.084	6.64e-05	–0.558	0.117	1.84e-06
Postcentral	–0.171	0.085	4.5e-02	–0.196	0.085	2.14e-02	–0.405	0.118	6.33e-04
Precentral	–0.207	0.083	1.21e-02	–0.356	0.083	1.79e-05	–0.553	0.115	1.61e-06
Precuneus	–0.195	0.084	2.03e-02	–0.314	0.084	2e-04	–0.479	0.117	4.42e-05
Superior frontal	–0.099	0.081	2.2e-01	–0.208	0.081	1.01e-02	–0.366	0.112	1.13e-03
Superior parietal	–0.21	0.081	9.34e-03	–0.251	0.081	1.91e-03	–0.331	0.112	3.23e-03
Superior temporal	–0.15	0.085	7.67e-02	–0.325	0.085	1.33e-04	–0.556	0.118	2.65e-06
Supramarginal	–0.135	0.085	1.14e-01	–0.176	0.086	3.99e-02	–0.336	0.119	4.93e-03
Temporal pole	–0.085	0.076	2.63e-01	–0.177	0.077	2.08e-02	–0.248	0.103	1.64e-02
Transverse temporal	–0.104	0.086	2.25e-01	–0.213	0.086	1.36e-02	–0.408	0.12	6.9e-04

## 4. Discussion

This study aimed to assess the relationship between deviations from typical brain development and psychopathology symptoms using three waves of neuroimaging and behavioral data from a large population-based cohort. We applied normative modeling to derive typical development curves, which showed regional differences in the development of subcortical and cortical volume, as well as cortical thickness and surface area. Psychopathology symptoms were related to deviations from typical development in subcortical volumes, and widespread regions across the cortex for cortical volume and surface area, and some regions for cortical thickness.

Our hypothesis driven and exploratory analyses together revealed that deviations from normative development related to psychopathology symptoms are not restricted to the a priori defined regions of interest, but rather that these deviations were present in regions across the entire cortex, as well as subcortical gray matter volumes. This raises the idea that the observed associations might not be region specific, but rather represent a global effect on brain development. Indeed, the majority of these findings did not remain after additional correction for ICV, indicating that the effects obtained are mostly global. Some areas, however, show a significant relationship on top of this global effect. Taken together, the observed pattern suggests that, while some regions may be particularly important for the emergence of psychopathology symptoms or affected by downstream effects of psychopathology (39), associations between cortical volume and psychopathology are not necessarily restricted to these regions. Given that emotion and behavior require integration of information that involves many brain regions, these small, but widespread differences may together lead to differences in psychopathology symptoms. Thus, our findings bolster the importance of analyzing the entire cortex and subcortex when assessing the relationship between brain morphology and psychopathology in youth, as opposed to restricting analyses to a priori defined regions of interest.

In line with our hypotheses, children with externalizing symptoms deviated from typical development for (sub-)cortical volumes in the ACC, OFC and amygdala, and children with DP symptoms deviated from typical development in the OFC, amygdala and striatum. As opposed to our initial hypothesis, we did not find evidence for a relationship between internalizing symptoms and the hypothesized regions of interest. Additionally, the relationship between deviations from typical development in the hippocampus and the striatum, and externalizing symptoms did not reach statistical significance. In line with the widespread alterations in development of subcortical and cortical volume, we additionally observed associations between the development of the ACC, and internalizing and DP symptoms, the rostral middle frontal cortex and externalizing and DP symptoms, and the hippocampus and DP symptoms. Earlier research on brain morphology and DP symptoms is relatively scarce, which is likely to explain why fewer regions had been reported to be related to DP symptoms previously. Our findings showed that associations between brain morphology and the DP were even more widespread than those for internalizing and externalizing symptoms. After correction for ICV, our results indicated region specific deviations from typical development for cortical volume in the ACC in relation to all psychopathology domains. Indeed, the ventral part of the ACC has been shown to have an important role in emotion regulation, including contextual fear generalization and the top-down regulation of aggressive impulses (49–52), and the dorsal ACC is crucial for cognitive control (53). Altered development of amygdala volume was only associated to externalizing and DP symptoms in this study, which is surprising given the role of the amygdala in processing of emotional cues that are important for all psychopathology domains (54). We had predicted that deviations in amygdala volume would also be associated with internalizing symptoms, although an earlier meta-analysis indicated that results on amygdala volume are somewhat inconsistent across studies, resulting in an absence of an effect in this meta-analysis (6). Regarding cortical thickness, deviations from typical development in the OFC were related to externalizing and DP symptoms, which is in line with the results obtained for cortical volume. However, in contrast with the findings for cortical volume, no associations between deviations from typical development in the ACC and any type of psychopathology were observed. Lastly, we hypothesized deviations from typical development in cortical thickness in the rostral middle frontal cortex to be related to internalizing symptoms, however, we did not find evidence for the presence of this relationship.

Deviations from typical development in surface area in children with symptoms of psychopathology showed remarkably similar results as those obtained for cortical volume. The overlap is likely partially explained by the high correlations between cortical volume and surface area, which in our sample ranged between 0.26 and 0.94. Given the low correlation between cortical thickness and surface area, these findings point toward surface area as an important brain morphology measure to study in relation to psychopathology. Although surface area is studied less extensively than cortical volume and cortical thickness in relation to psychopathology, recent work also showed similar results between psychopathology, and both cortical volume and surface area, but no relationship with cortical thickness (55). Contrary to these findings and the current findings, other work has observed alterations in cortical thickness in relation to multiple domains of psychopathology, whereas alterations in surface area were specific to externalizing disorders (56). A critical difference is, however, that the latter study evaluated the association between childhood psychopathology and brain morphology in mid-adulthood, and thus may not generalize to developmental populations. It will be important for future work to extend the current findings by assessing the relationship between brain morphology and psychopathology at multiple ages across the lifespan.

In both our hypothesis driven and exploratory analyses we showed that deviations from normative development were associated with psychopathology. Although the overlap in confidence intervals in the majority of regions do not suggest significant differences in effect sizes between psychopathology domains, a consistent pattern is observed with the largest effect sizes for DP symptoms and the smallest effect sizes for internalizing symptoms. This pattern was also observed in earlier work using the first and third wave of the current sample, for the relationship between cognitive performance, and internalizing, externalizing and DP symptoms (57, 58). These findings align closely with recent evidence that some alterations in brain structure and function are shared across many psychiatric disorders (59, 60). It is likely that the overlap in involved brain regions can partially be attributed to the high correlation among psychopathology domains. For example, internalizing and externalizing symptoms generally correlate with a coefficient of around 0.5 (61). Achenbach et al. (61) recommended adjustment for externalizing symptoms when internalizing symptoms are assessed and vice versa. Following this recommendation we performed sensitivity analyses for those regions in which deviations from typical development were related to multiple psychopathology domains. Our findings indicated that only DP symptoms were related to regional deviations from typical development above and beyond internalizing and externalizing symptoms. Thus, our findings add to the current knowledge that those with DP symptoms are most heavily affected in terms of symptomatology (62) and cognitive performance (57, 58), that they are also most heavily affected in terms of deviations from brain development. A promising line of research that has emerged in recent years, aims to unpack the shared variance between individual dimensions of psychopathology into a general psychopathology factor (63, 64). The variance in symptomatology that remains after extraction of the general psychopathology factor can then be viewed as more specific internalizing and externalizing symptoms. Indeed, recent work has used a similar approach to normative modeling as used in the current study, and showed that general psychopathology was associated with deviations from typical development for gray matter volume in widespread regions across the cortex, and additionally identified regions that associated with specific psychopathology dimensions (17). This study focused on gray matter volume and used clinical cases to assess associations with psychopathology. Thus, a promising extension of our study would be to assess whether deviations from typical development in relation to the general psychopathology factor are also more pronounced in cortical volume than cortical thickness.

Regarding the direction of effect, negative deviations from typical development were observed for cortical volume and surface area, and psychopathology, whereas positive deviations were observed in the relationship between cortical thickness and psychopathology. These directions were consistent across all psychopathology domains, in which very few interaction effects for age were observed. The absence of this interaction effect suggests that the direction of effect between psychopathology symptoms and deviations from typical development were largely stable between 6 and 16 years of age. Thus, while we had hypothesized an age-dependent effect on the deviations of typical development related to internalizing symptoms, we only provide evidence for age related effects in the development of the fusiform and parahippocampal volume in relation to externalizing symptoms. To provide more context to the direction of effect, both in cortical volume and thickness, it would be beneficial to not only establish typical development curves using cross-sectional data, but also using longitudinal MRI data. Indeed, some evidence suggests that longitudinal trajectories of cortical development are different in those with internalizing (1, 8) and externalizing symptoms (24). Extending findings of the current study, by studying temporal changes in the deviations from typical development in relation to temporal changes in psychopathology, could provide unique insights in the bidirectional relationship between behavioral and brain development.

Findings from our normative model are in line with contemporary work that finds the average growth trajectory for gray matter volume to peak at 6–10 years of age, after which it declines (12, 65–67). This pattern is also observed for total brain volume, although earlier work did not model a decline in total brain volume after this peak (68) . The parietal and occipital lobe mirror this inverted U-shaped pattern in our normative model. Regional differences in developmental trajectories have been reported previously (11, 13, 69) and indeed we extend prior knowledge by showing that, similar to the earlier work (69), across a span of 6–16 years of age, many regions in the frontal and temporal lobe reach their peak volume after 6 years of age. Thus, our findings indicate that each brain region develops at its own pace, with brain regions located in the same lobe showing similar developmental trajectories. We also observed almost identical patterns in subcortical gray matter volume as previous work (12, 66), which showed inverted U-shaped trajectories that reached a peak around 15 years of age. Our findings indicate either an inverted U-shaped or positive developmental trajectory for individual subcortical regions. Further, our model showed that cortical thickness has a negative developmental curve for the vast majority of regions, with only some regions showing a fairly flat trajectory. This is consistent with earlier findings showing a decrease in cortical thickness across this age range (70) and largely consistent with other work, although the peak of cortical thickness was either reached at earlier (66) ages for average cortical thickness or later ages for average as well as regional cortical thickness (65, 71). Further, our findings showed that the rate of change differs across brain regions. For example, regions in the occipital lobe have a steep decline in cortical thickness between 6 and 12 years of age, after which they reach a plateau, whereas regions in the parietal and cingulate cortex have a more linear decline over time, of which most do not yet reach a plateau at 16 years of age. Regarding surface area, earlier work indicated that across the entire cortex, development follows an inverted U-shaped trajectory across childhood and adolescence (65, 66). In line with these findings, our results indicate similar trajectories for most regions. However, our findings also indicate that for regions mostly in the frontal and temporal lobe, surface area continues to increase until after 10–12 years of age, mirroring the regional differences in development observed for cortical volume and thickness.

Our study has several strengths and limitations. First, a key strength of the current study is that we used normative modeling to establish z-scores representing deviations from normative development, which were subsequently related to psychopathology symptoms. Earlier work has shown that these regional deviations form typical development provide a greater prediction accuracy for psychopathology symptoms than using raw measures of brain morphology (17). Second, this study included a large age range, spanning early childhood to mid-adolescence, which allowed us to extend contemporary findings (12, 70) in an age range that has not been studied extensively. Third, we were able to adjust our analyses for many potentially confounding factors. Although we interpreted the second model, some associations observed in model 1, corrected for biological sex and handedness, attenuated upon adjustment for SES and child national origin. These differences in the results obtained from the first and second model indicate the importance of adjustment for potentially confounding factors. Fourth, the population-based setting of the current study is both a strength and limitation. To derive typical developmental trajectories of brain morphology, a population-based sample is ideal. However, the majority of participants in this study exhibit relatively low levels of psychopathology, limiting the power to detect associations that might exist in those with clinical psychopathology levels. Fifth, while the current sample covers a large and important age range for developmental studies, the data acquired was not equally distributed across all ages. This can potentially influence the results around ages where fewer data was available. Sixth, although the current findings were derived from one of the largest population-based samples covering childhood and adolescence, our findings warrant replication in other comparable cohorts. Seventh, the data at T1 was obtained on a different MRI scanner than the data at T2 and T3, which may have influenced the results. Finally, a limitation of the current study is the amount of inter-individual variability in brain morphology measures and subsequently in the obtained z-scores. This results in small effect sizes in the associations we observed. However, small effect sizes are consistently reported for studies on brain morphology and psychopathology, and in those that have obtained large effect sizes, the effect size is often inflated by the small sample size (72).

In summary, this study charted regional typical development of subcortical and cortical volume, surface area and cortical thickness. Findings showed that deviations from this typical development curve were related to psychopathology symptoms in widespread regions of the cerebral (sub-)cortex. DP symptoms were related to regional deviations from typical brain development above and beyond internalizing and externalizing symptoms in cortical volume. Our findings underline the evidence that assessing deviations from typical development in terms of (sub-)cortical volume and thickness can provide insights in the coupling between brain and behavioral development.

## Data Availability Statement

The datasets presented in this article are not readily available because Generation R data is available to researchers upon reasonable request. Requests should be directed to the management team of the Generation R Study. Individual level data are not publicly available for privacy and ethical restrictions. Requests to access the datasets should be directed to generationr@erasmusmc.

## Ethics Statement

The studies involving human participants were reviewed and approved by METC Erasmus MC. Written informed consent to participate in this study was provided by the participants' legal guardian/next of kin.

## Author Contributions

EAWG: methodology, writing–original draft, and writing–review and editing. EB: conceptualization, data curation, formal analysis, methodology, visualization, writing–original draft, and writing–review and editing. EPTG: formal analysis, codebase maintenance, methodology, visualization, writing–original draft, and writing–review and editing. TW: conceptualization, funding acquisition, methodology, project administration, resources, supervision, and writing–review and editing. VC: methodology, resources, and writing–review and editing. All authors contributed to the article and approved the submitted version.

## Funding

This work was supported by the Sophia Children's Hospital Research Foundation (SSWO) Project #S18-68, #S20-48 and the Netherlands Organization for Health Research and Development (ZonMw) TOP Project Number 91211021. The general design of the Generation R Study is made possible by financial support from the Erasmus Medical Center, Rotterdam, ZonMw, the Netherlands Organization for Scientific Research (NWO), and the Ministry of Health, Welfare and Sport, and is conducted by the Erasmus Medical Center in close collaboration with the Faculty of Social Sciences of the Erasmus University Rotterdam, and the Stichting Trombosedienst & Artsenlaboratorium Rijnmond (STAR-MDC), Rotterdam.

## Conflict of Interest

The authors declare that the research was conducted in the absence of any commercial or financial relationships that could be construed as a potential conflict of interest.

## Publisher's Note

All claims expressed in this article are solely those of the authors and do not necessarily represent those of their affiliated organizations, or those of the publisher, the editors and the reviewers. Any product that may be evaluated in this article, or claim that may be made by its manufacturer, is not guaranteed or endorsed by the publisher.
