# Supplementary material for: Neurodevelopmental Trajectories in Children With Internalizing, Externalizing and Emotion Dysregulation Symptoms
Source: Front Psychiatry. 2022 Mar 18;13:846201. doi: 10.3389/fpsyt.2022.846201 (PMC8974911; doi:10.3389/fpsyt.2022.846201)
Supplement: Supplementary file 1 [file Data_Sheet_1.PDF]

Supplementary Material

1 SUPPLEMENTARY DATA

1.1 Deviations from preregistration

Hypotheses and analyses for this project were publicly preregistered, a time-stamped version of this preregistration is available via: `www.osf.io/aqc4s`. We have deviated from this preregistration, in four ways. First, while we originally stated that linear regression analyses would be used to assess the relationship between deviations from typical development and psychopathology, we have performed a linear mixed model to analyze the data. We have altered the analyses, given that our dataset included repeated measurements for some participants. The linear mixed model can account for within participant clustering of the data. Second, an analysis was added to explore age-related differences in the slope of the relationship, which was done by including an interaction term between z-scores, representing the deviations from typical development, and age at MRI scan. Finally, two sensitivity and one set of post hoc analyses were added to assess the robustness of our findings. In the first sensitivity analysis we corrected our analyses with (sub-)cortical volume for total intracranial volume (ICV) to assess whether the observed effects were global or region specific. In the second sensitivity analysis we simultaneously modelled all psychopathology domains that showed a significant relationship with regional deviations from typical development to assess whether individual psychopathology domains were related to brain development after correction for other psychopathology domains. In the set of post hoc analyses, normative developmental trajectories were modeled for surface area, and deviations from typical development were related to psychopathology measures.

2 SUPPLEMENTARY TABLES AND FIGURES

2.1 Tables

Table S1. Hypothesis driven (sub-)cortical volume model 1

Brain region	Psychopathology symptoms	B	S.E.	p-value
Anterior cingulate	Internalizing	-0.382	0.086	9.22e-06
	Externalizing	-0.455	0.086	1.34e-07
	Dysregulation Profile	-0.69	0.119	8.79e-09
Orbitofrontal	Internalizing	-0.232	0.083	5.14e-03
	Externalizing	-0.38	0.083	5.17e-06
	Dysregulation Profile	-0.51	0.114	8.12e-06
Rostral middle frontal	Internalizing	-0.306	0.085	3.03e-04
	Externalizing	-0.385	0.085	5.85e-06
	Dysregulation Profile	-0.604	0.116	2.44e-07
Amygdala	Internalizing	-0.247	0.078	1.48e-03
	Externalizing	-0.409	0.078	1.7e-07
	Dysregulation Profile	-0.468	0.107	1.26e-05
Hippocampus	Internalizing	-0.186	0.085	2.86e-02
	Externalizing	-0.249	0.085	3.39e-03
	Dysregulation Profile	-0.47	0.118	6.87e-05
Striatum	Internalizing	-0.257	0.086	2.85e-03
	Externalizing	-0.227	0.086	8.45e-03
	Dysregulation Profile	-0.414	0.12	5.54e-04

Table S2. Hypothesis driven cortical thickness model 1

Brain region	Psychopathology symptoms	B	S.E.	p-value
Anterior cingulate	Internalizing	0.03	0.073	6.85e-01
	Externalizing	0.085	0.073	2.46e-01
	Dysregulation Profile	0.189	0.1	5.96e-02
Orbitofrontal	Internalizing	0.053	0.056	3.49e-01
	Externalizing	0.128	0.056	2.3e-02
	Dysregulation Profile	0.168	0.076	2.71e-02
Rostral middle frontal	Internalizing	0.046	0.064	4.73e-01
	Externalizing	0.091	0.065	1.58e-01
	Dysregulation Profile	0.086	0.087	3.21e-01

Table S3. Exploratory (sub-)cortical volume model 1

	Internalizing			Externalizing			Dysregulation Profile		
Brain region	B	S.E.	p-value	B	S.E.	p-value	B	S.E.	p-value
Bankssts	-0.183	0.085	3.26e-02	-0.285	0.085	8.73e-04	-0.366	0.118	1.99e-03
Caudal middle frontal	-0.219	0.086	1.07e-02	-0.24	0.086	5.03e-03	-0.44	0.118	2.1e-04
Cuneus	-0.31	0.083	1.96e-04	-0.358	0.083	1.68e-05	-0.602	0.115	1.92e-07
Entorhinal	-0.087	0.081	2.82e-01	-0.123	0.081	1.3e-01	-0.175	0.11	1.11e-01
Frontal pole	0.048	0.07	4.9e-01	0.053	0.071	4.53e-01	0.084	0.094	3.71e-01
Fusiform	-0.26	0.086	2.43e-03	-0.384	0.086	8.5e-06	-0.499	0.118	2.64e-05
Inferior parietal	-0.283	0.084	8.06e-04	-0.433	0.084	3.27e-07	-0.61	0.117	2.05e-07
Inferior temporal	-0.233	0.084	5.44e-03	-0.352	0.084	2.83e-05	-0.497	0.115	1.63e-05
Insula	-0.25	0.085	3.25e-03	-0.319	0.085	1.84e-04	-0.53	0.117	6.26e-06
Isthmus cingulate	-0.302	0.085	3.79e-04	-0.382	0.085	7.25e-06	-0.581	0.118	9.78e-07
Lateral occipital	-0.372	0.083	8.58e-06	-0.434	0.083	2.14e-07	-0.68	0.116	5.12e-09
Lingual	-0.281	0.082	6.42e-04	-0.34	0.082	3.6e-05	-0.568	0.114	7.05e-07
Middle temporal	-0.272	0.085	1.32e-03	-0.395	0.085	3.3e-06	-0.562	0.116	1.46e-06
Paracentral	-0.155	0.084	6.59e-02	-0.309	0.084	2.45e-04	-0.363	0.116	1.82e-03
Parahippocampal	-0.033	0.086	7.02e-01	-0.225	0.086	8.82e-03	-0.186	0.118	1.17e-01
Pars opercularis	-0.152	0.086	7.6e-02	-0.212	0.086	1.35e-02	-0.391	0.119	1.03e-03
Pars orbitalis	-0.194	0.085	2.31e-02	-0.308	0.086	3.3e-04	-0.396	0.117	7.46e-04
Pars triangularis	-0.223	0.085	8.39e-03	-0.261	0.085	2.12e-03	-0.474	0.117	5.57e-05
Pericalcarine	-0.132	0.082	1.07e-01	-0.19	0.082	2.06e-02	-0.408	0.113	3.07e-04
Posterior cingulate	-0.208	0.085	1.45e-02	-0.344	0.085	5.36e-05	-0.563	0.118	2.05e-06
Postcentral	-0.252	0.083	2.56e-03	-0.343	0.083	4.12e-05	-0.527	0.116	5.92e-06
Precentral	-0.256	0.086	3.14e-03	-0.382	0.086	1.04e-05	-0.587	0.12	1.05e-06
Precuneus	-0.387	0.083	3.23e-06	-0.475	0.083	1.2e-08	-0.63	0.115	5.02e-08
Superior frontal	-0.161	0.085	5.65e-02	-0.249	0.085	3.36e-03	-0.353	0.117	2.67e-03
Superior parietal	-0.332	0.084	7.55e-05	-0.405	0.084	1.47e-06	-0.521	0.116	7.38e-06
Superior temporal	-0.148	0.085	8.11e-02	-0.253	0.085	2.88e-03	-0.425	0.117	2.85e-04
Supramarginal	-0.206	0.085	1.54e-02	-0.213	0.085	1.23e-02	-0.359	0.118	2.38e-03
Temporal pole	-0.042	0.076	5.85e-01	-0.064	0.077	4.06e-01	-0.053	0.102	6.01e-01
Transverse temporal	-0.131	0.085	1.22e-01	-0.219	0.085	9.79e-03	-0.325	0.118	5.88e-03
Pallidum	-0.088	0.08	2.72e-01	-0.021	0.08	7.89e-01	-0.166	0.109	1.3e-01
Thalamus	-0.299	0.085	4.79e-04	-0.357	0.086	3.21e-05	-0.561	0.118	2.36e-06

Table S4. Exploratory cortical thickness model 1

	Internalizing			Externalizing			Dysregulation Profile		
Brain region	B	S.E.	p-value	B	S.E.	p-value	B	S.E.	p-value
Bankssts	-0.128	0.075	9.01e-02	0.048	0.076	5.24e-01	0.085	0.104	4.15e-01
Caudal middle frontal	0.032	0.08	6.91e-01	0.015	0.081	8.57e-01	-0.027	0.109	8.06e-01
Cuneus	-0.013	0.065	8.44e-01	-0.044	0.065	5.03e-01	-0.077	0.089	3.89e-01
Entorhinal	-0.092	0.079	2.42e-01	0.023	0.079	7.68e-01	-0.076	0.106	4.7e-01
Frontalpole	0.135	0.064	3.57e-02	0.197	0.065	2.44e-03	0.278	0.086	1.29e-03
Fusiform	-0.023	0.074	7.54e-01	0.079	0.074	2.89e-01	0.053	0.101	5.99e-01
Inferior parietal	-0.134	0.069	5.23e-02	-0.152	0.07	2.92e-02	-0.177	0.094	5.99e-02
Inferior temporal	0.059	0.074	4.25e-01	0.108	0.074	1.47e-01	0.084	0.1	4.03e-01
Insula	0.01	0.071	8.87e-01	0.072	0.071	3.13e-01	0.08	0.096	4.05e-01
Isthmus cingulate	-0.071	0.082	3.88e-01	-0.071	0.083	3.9e-01	-0.03	0.114	7.95e-01
Lateral occipital	0.008	0.062	8.92e-01	-0.093	0.062	1.35e-01	-0.1	0.084	2.34e-01
Lingual	-0.064	0.057	2.6e-01	-0.03	0.057	6.03e-01	-0.084	0.078	2.81e-01
Middle temporal	-0.016	0.076	8.34e-01	0.108	0.076	1.57e-01	0.084	0.103	4.15e-01
Paracentral	-0.072	0.069	3.01e-01	-0.057	0.07	4.13e-01	-0.04	0.095	6.74e-01
Parahippocampal	-0.122	0.086	1.57e-01	-0.095	0.087	2.71e-01	-0.116	0.119	3.33e-01
Pars opercularis	0.038	0.072	5.97e-01	0.052	0.073	4.73e-01	0.059	0.099	5.49e-01
Pars orbitalis	0.108	0.074	1.45e-01	0.121	0.075	1.07e-01	0.208	0.101	3.89e-02
Pars triangularis	0.052	0.066	4.31e-01	0.075	0.066	2.56e-01	0.143	0.09	1.12e-01
Pericalcarine	0.132	0.06	2.7e-02	0.112	0.06	6.31e-02	0.067	0.081	4.07e-01
Posterior cingulate	0.093	0.07	1.85e-01	0.184	0.07	8.78e-03	0.23	0.096	1.68e-02
Postcentral	-0.028	0.074	7.02e-01	-0.134	0.074	7.2e-02	-0.129	0.101	2.03e-01
Precentral	-0.021	0.079	7.95e-01	-0.05	0.079	5.29e-01	-0.043	0.107	6.9e-01
Precuneus	-0.164	0.066	1.35e-02	-0.109	0.066	1.02e-01	-0.068	0.091	4.55e-01
Superior frontal	0.103	0.073	1.59e-01	0.132	0.073	7.09e-02	0.21	0.099	3.42e-02
Superior parietal	-0.07	0.069	3.06e-01	-0.149	0.069	3.12e-02	-0.161	0.093	8.52e-02
Superior temporal	0.079	0.074	2.9e-01	0.179	0.075	1.68e-02	0.213	0.101	3.58e-02
Supramarginal	-0.057	0.071	4.21e-01	-0.024	0.071	7.37e-01	0.028	0.097	7.72e-01
Temporal pole	0.017	0.076	8.2e-01	0.072	0.076	3.48e-01	0.102	0.101	3.15e-01
Transverse temporal	0.068	0.079	3.87e-01	0.117	0.079	1.39e-01	0.212	0.108	5.05e-02

Table S5. Interaction effect age: Hypothesis driven (sub-)cortical volume model 2

Brain region	Psychopathology symptoms	B	S.E.	p-value
Anterior cingulate	Internalizing	-0.014	0.029	6.33e-01
	Externalizing	0.036	0.028	2.04e-01
	Dysregulation Profile	-0.005	0.037	9e-01
Orbitofrontal	Internalizing	-0.029	0.029	3.25e-01
	Externalizing	0.041	0.029	1.55e-01
	Dysregulation Profile	0.022	0.038	5.54e-01
Rostral middle frontal	Internalizing	-0.055	0.029	5.59e-02
	Externalizing	0.02	0.028	4.82e-01
	Dysregulation Profile	-0.028	0.037	4.44e-01
Amygdala	Internalizing	-0.028	0.027	3.01e-01
	Externalizing	0.069	0.027	9.84e-03
	Dysregulation Profile	0.042	0.035	2.39e-01
Hippocampus	Internalizing	-0.044	0.028	1.2e-01
	Externalizing	0.01	0.028	7.33e-01
	Dysregulation Profile	0.006	0.037	8.81e-01
Striatum	Internalizing	-0.035	0.029	2.22e-01
	Externalizing	0.059	0.028	3.68e-02
	Dysregulation Profile	0.019	0.037	6.04e-01

*Indicates significance after correction for multiple testing using FDR-BH at a q-value of 0.05

Table S6. Interaction effect age: Hypothesis driven cortical thickness model 2

Brain region	Psychopathology symptoms	B	S.E.	p-value
Anterior cingulate	Internalizing	-0.045	0.026	7.57e-02
	Externalizing	-0.04	0.025	1.11e-01
	Dysregulation Profile	-0.054	0.033	1.05e-01
Orbitofrontal	Internalizing	-0.033	0.02	1.01e-01
	Externalizing	-0.041	0.02	4.38e-02
	Dysregulation Profile	-0.034	0.027	1.99e-01
Rostral middle frontal	Internalizing	-0.005	0.024	8.27e-01
	Externalizing	-0.009	0.023	6.91e-01
	Dysregulation Profile	0.007	0.031	8.26e-01

*Indicates significance after correction for multiple testing using FDR-BH at a q-value of 0.05

Table S7. Interaction effect age: Exploratory (sub-)cortical volume model 2

	Internalizing			Externalizing				Dysregulation Profile		
Brain region	B	S.E.	p-value	B	S.E.	p-value		B	S.E.	p-value
Bankssts	-0.035	0.029	2.3e-01	0.031	0.029	2.86e-01		0.014	0.038	7.07e-01
Caudal middle frontal	-0.059	0.029	4.57e-02	0.007	0.029	7.97e-01		-0.015	0.038	6.95e-01
Cuneus	0.013	0.028	6.39e-01	0.039	0.027	1.54e-01		0.034	0.036	3.38e-01
Entorhinal	0.01	0.027	7.25e-01	0.051	0.027	6.02e-02		0.037	0.035	2.97e-01
Frontal pole	-0.055	0.029	5.73e-02	-0.041	0.029	1.49e-01		-0.024	0.038	5.28e-01
Fusiform	-0.008	0.029	7.86e-01	0.099	0.028	4.49e-04	*	0.058	0.037	1.15e-01
Inferior parietal	-0.01	0.028	7.22e-01	0.053	0.028	5.45e-02		0.048	0.036	1.88e-01
Inferior temporal	-0.028	0.027	3.1e-01	0.024	0.027	3.84e-01		-0.011	0.036	7.64e-01
Insula	-0.064	0.029	2.42e-02	0.006	0.028	8.23e-01		-0.05	0.037	1.74e-01
Isthmus cingulate	-0.027	0.028	3.4e-01	0.043	0.028	1.21e-01		0.016	0.037	6.62e-01
Lateral occipital	-0.024	0.028	3.88e-01	0.029	0.027	2.82e-01		0.013	0.036	7.16e-01
Lingual	0.014	0.027	6.06e-01	0.033	0.027	2.26e-01		0.004	0.036	9.13e-01
Middle temporal	-0.04	0.028	1.65e-01	0.007	0.028	7.98e-01		-0.025	0.037	4.99e-01
Paracentral	-0.019	0.029	4.96e-01	0.008	0.028	7.7e-01		0.007	0.037	8.45e-01
Parahippocampal	0.022	0.028	4.42e-01	0.101	0.028	3.17e-04	*	0.072	0.037	5.06e-02
Pars opercularis	-0.007	0.028	8.08e-01	0.05	0.028	7.29e-02		0.048	0.037	1.9e-01
Pars orbitalis	-0.032	0.028	2.67e-01	0.036	0.028	1.97e-01		0.005	0.037	8.95e-01
Pars triangularis	-0.045	0.029	1.22e-01	0.006	0.029	8.24e-01		-0.012	0.038	7.41e-01
Pericalcarine	0.041	0.027	1.28e-01	0.05	0.027	6.31e-02		0.047	0.035	1.76e-01
Posterior cingulate	0.047	0.028	9.47e-02	0.071	0.028	1.09e-02		0.074	0.036	4.16e-02
Postcentral	-0.024	0.027	3.85e-01	0.023	0.027	4.06e-01		0.025	0.036	4.75e-01
Precentral	-0.059	0.028	3.55e-02	0.011	0.028	6.95e-01		-0.033	0.036	3.63e-01
Precuneus	0.006	0.028	8.43e-01	0.06	0.028	3.03e-02		0.036	0.036	3.18e-01
Superior frontal	-0.06	0.028	3.05e-02	0.007	0.028	7.87e-01		-0.005	0.036	8.95e-01
Superior parietal	0.005	0.028	8.62e-01	0.064	0.027	1.92e-02		0.051	0.036	1.54e-01
Superior temporal	-0.045	0.029	1.16e-01	0.041	0.028	1.45e-01		-0.001	0.037	9.82e-01
Supramarginal	-0.046	0.028	1.03e-01	0.046	0.028	9.7e-02		0.016	0.037	6.56e-01
Temporal pole	-0.011	0.03	7.14e-01	0.025	0.03	4.06e-01		-0.001	0.04	9.7e-01
Transverse temporal	-0.032	0.028	2.49e-01	0.035	0.028	2.02e-01		0.032	0.036	3.8e-01
Pallidum	-0.062	0.028	2.39e-02	0.013	0.027	6.38e-01		-0.028	0.036	4.31e-01
Thalamus	-0.07	0.029	1.41e-02	0.011	0.028	7.1e-01		-0.03	0.037	4.14e-01

*Indicates significance after correction for multiple testing using FDR-BH at a q-value of 0.05

Table S8. Interaction effect age: Exploratory cortical thickness model 2

	Internalizing			Externalizing			Dysregulation Profile		
Brain region	B	S.E.	p-value	B	S.E.	p-value	B	S.E.	p-value
Bankssts	-0.04	0.026	1.31e-01	-0.023	0.026	3.87e-01	-0.031	0.034	3.59e-01
Caudal middle frontal	-0.062	0.029	3.6e-02	-0.03	0.029	3.04e-01	-0.032	0.038	3.99e-01
Cuneus	-0.015	0.023	5.22e-01	-0.011	0.023	6.29e-01	-0.023	0.03	4.56e-01
Entorhinal	0.001	0.03	9.65e-01	0.046	0.03	1.2e-01	0.007	0.039	8.55e-01
Frontal pole	-0.022	0.027	4.13e-01	-0.064	0.027	1.77e-02	-0.024	0.036	5.06e-01
Fusiform	-0.036	0.027	1.79e-01	-0.007	0.026	7.83e-01	-0.033	0.035	3.35e-01
Inferior parietal	-0.043	0.026	9.57e-02	-0.002	0.026	9.42e-01	-0.009	0.034	8.02e-01
Inferior temporal	-0.041	0.027	1.36e-01	-0.004	0.027	8.93e-01	-0.024	0.036	5.1e-01
Insula	-0.039	0.026	1.38e-01	0.009	0.026	7.25e-01	0.018	0.034	6.03e-01
Isthmus cingulate	-0.021	0.028	4.58e-01	-0.002	0.028	9.45e-01	-0.036	0.036	3.18e-01
Lateral occipital	-0.029	0.023	2.13e-01	-0.032	0.023	1.6e-01	-0.043	0.03	1.57e-01
Lingual	-0.014	0.02	4.82e-01	-0.007	0.02	7.47e-01	-0.021	0.027	4.24e-01
Middle temporal	-0.042	0.028	1.33e-01	-0.02	0.028	4.71e-01	-0.057	0.037	1.17e-01
Paracentral	-0.044	0.025	7.94e-02	-0.058	0.025	1.86e-02	-0.056	0.033	8.74e-02
Parahippocampal	-0.008	0.029	7.75e-01	0.013	0.029	6.56e-01	0.006	0.038	8.72e-01
Pars opercularis	-0.037	0.026	1.57e-01	-0.024	0.026	3.5e-01	-0.019	0.034	5.88e-01
Pars orbitalis	-0.018	0.027	5.15e-01	-0.002	0.027	9.48e-01	0.006	0.035	8.71e-01
Pars triangularis	-0.028	0.024	2.38e-01	-0.023	0.024	3.37e-01	-0.006	0.031	8.39e-01
Pericalcarine	0.005	0.022	8.31e-01	0.024	0.021	2.65e-01	0.021	0.028	4.5e-01
Posterior cingulate	0.008	0.025	7.61e-01	-0.016	0.025	5.17e-01	0.006	0.033	8.6e-01
Postcentral	0.028	0.026	2.92e-01	0.035	0.026	1.75e-01	0.06	0.034	8.23e-02
Precentral	-0.064	0.029	2.56e-02	-0.013	0.028	6.48e-01	-0.033	0.037	3.76e-01
Precuneus	-0.016	0.023	4.88e-01	-0.015	0.023	5.16e-01	-0.014	0.03	6.28e-01
Superior frontal	-0.037	0.026	1.59e-01	-0.024	0.026	3.56e-01	-0.009	0.034	7.9e-01
Superior parietal	-0.013	0.025	6.1e-01	-0.002	0.025	9.52e-01	-0.004	0.033	9.1e-01
Superior temporal	-0.029	0.027	2.77e-01	0.015	0.027	5.85e-01	-0.012	0.035	7.27e-01
Supramarginal	-0.015	0.025	5.52e-01	0.005	0.025	8.31e-01	0.011	0.033	7.43e-01
Temporal pole	0.004	0.03	9.06e-01	0.032	0.03	2.93e-01	0.016	0.04	6.92e-01
Transverse temporal	-0.015	0.028	5.94e-01	0.013	0.028	6.49e-01	0.026	0.036	4.72e-01

*Indicates significance after correction for multiple testing using FDR-BH at a q-value of 0.05

Table S9. Hypothesis driven (sub-)cortical volume, corrected for ICV

Brain region	Psychopathology symptoms	B	S.E.	p-value
Anterior cingulate	Internalizing	-0.167	0.08	3.71e-02
	Externalizing	-0.206	0.08	1.03e-02
	Dysregulation Profile	-0.314	0.111	4.78e-03
Orbitofrontal	Internalizing	0.034	0.074	6.46e-01
	Externalizing	-0.097	0.074	1.91e-01
	Dysregulation Profile	-0.096	0.1	3.41e-01
Rostral middle frontal	Internalizing	-0.066	0.077	3.96e-01
	Externalizing	-0.108	0.078	1.64e-01
	Dysregulation Profile	-0.203	0.106	5.53e-02
Amygdala	Internalizing	-0.053	0.08	5.11e-01
	Externalizing	-0.208	0.081	1.01e-02
	Dysregulation Profile	-0.122	0.11	2.66e-01
Hippocampus	Internalizing	0.049	0.083	5.54e-01
	Externalizing	0.024	0.084	7.76e-01
	Dysregulation Profile	-0.059	0.116	6.12e-01
Striatum	Internalizing	-0.059	0.083	4.76e-01
	Externalizing	0.028	0.083	7.41e-01
	Dysregulation Profile	-0.019	0.116	8.67e-01

Table S10. Exploratory (sub-)cortical volume, corrected for ICV

	Internalizing			Externalizing			Dysregulation Profile		
Brain region	B	S.E.	p-value	B	S.E.	p-value	B	S.E.	p-value
Bankssts	-0.013	0.081	8.72e-01	-0.096	0.081	2.4e-01	-0.088	0.112	4.34e-01
Caudal middle frontal	0.027	0.082	7.44e-01	0.043	0.083	6.05e-01	-0.033	0.114	7.69e-01
Cuneus	-0.151	0.079	5.45e-02	-0.182	0.079	2.09e-02	-0.372	0.109	6.92e-04
Entorhinal	0.039	0.08	6.28e-01	0.031	0.08	7.03e-01	0.022	0.109	8.37e-01
Frontal pole	0.086	0.07	2.2e-01	0.088	0.071	2.12e-01	0.108	0.094	2.52e-01
Fusiform	0.036	0.082	6.56e-01	-0.012	0.082	8.87e-01	-0.046	0.112	6.81e-01
Inferior parietal	-0.059	0.075	4.35e-01	-0.193	0.076	1.11e-02	-0.263	0.105	1.2e-02
Inferior temporal	0.016	0.083	8.48e-01	-0.088	0.083	2.92e-01	-0.098	0.113	3.87e-01
Insula	-0.004	0.078	9.54e-01	-0.053	0.078	4.93e-01	-0.13	0.106	2.21e-01
Isthmus cingulate	-0.087	0.078	2.63e-01	-0.135	0.078	8.21e-02	-0.226	0.108	3.67e-02
Lateral occipital	-0.147	0.075	5.18e-02	-0.197	0.076	9.27e-03	-0.334	0.105	1.47e-03
Lingual	-0.167	0.076	2.83e-02	-0.204	0.076	7.64e-03	-0.361	0.106	6.68e-04
Middle temporal	-0.032	0.082	6.95e-01	-0.145	0.083	8.14e-02	-0.179	0.113	1.13e-01
Paracentral	0.076	0.076	3.18e-01	-0.053	0.076	4.92e-01	-0.004	0.105	9.7e-01
Parahippocampal	0.124	0.084	1.4e-01	-0.039	0.084	6.44e-01	0.095	0.116	4.12e-01
Pars opercularis	0.048	0.081	5.54e-01	0	0.081	9.96e-01	-0.075	0.112	5.03e-01
Pars orbitalis	-0.015	0.082	8.51e-01	-0.114	0.083	1.7e-01	-0.12	0.113	2.88e-01
Pars triangularis	-0.071	0.081	3.82e-01	-0.09	0.081	2.67e-01	-0.232	0.112	3.84e-02
Pericalcarine	-0.052	0.079	5.11e-01	-0.097	0.079	2.18e-01	-0.28	0.109	1.02e-02
Posterior cingulate	-0.001	0.078	9.93e-01	-0.112	0.078	1.5e-01	-0.216	0.108	4.59e-02
Postcentral	0.003	0.074	9.64e-01	-0.064	0.074	3.88e-01	-0.129	0.102	2.07e-01
Precentral	-0.032	0.08	6.86e-01	-0.155	0.08	5.36e-02	-0.201	0.111	6.87e-02
Precuneus	-0.117	0.072	1.04e-01	-0.176	0.072	1.51e-02	-0.209	0.1	3.67e-02
Superior frontal	0.138	0.077	7.38e-02	0.083	0.077	2.81e-01	0.147	0.106	1.66e-01
Superiorparietal	-0.089	0.074	2.33e-01	-0.138	0.075	6.41e-02	-0.139	0.103	1.76e-01
Superior temporal	0.054	0.078	4.9e-01	-0.035	0.078	6.55e-01	-0.077	0.107	4.71e-01
Supramarginal	0.059	0.076	4.43e-01	0.09	0.077	2.38e-01	0.053	0.106	6.17e-01
Temporal pole	-0.006	0.076	9.33e-01	-0.022	0.077	7.78e-01	0.005	0.102	9.59e-01
Transverse temporal	0.079	0.079	3.16e-01	0.003	0.08	9.72e-01	-0.006	0.11	9.55e-01
Pallidum	0.04	0.081	6.17e-01	0.146	0.081	7.33e-02	0.137	0.11	2.15e-01
Thalamus	-0.032	0.08	6.87e-01	-0.05	0.081	5.39e-01	-0.087	0.111	4.34e-01

Table S11. Simultaneously modelled significant psychpathology domains in relation to deviations from typical development for (sub-)cortical volume

Brain region	Psychopathology symptoms	Psychopathology symptoms modelled simultaneously	B	S.E.	p-value
Anterior cingulate	Internalizing	Internalizing, Externalizing & DP	0.062	0.052	2.38e-01
Anterior cingulate	Externalizing	Internalizing, Externalizing & DP	0.045	0.038	2.32e-01
Anterior cingulate	Dysregulation Profile	Internalizing, Externalizing & DP	-0.154	0.042	3.09e-04
Precuneus	Internalizing	Internalizing, Externalizing & DP	0.045	0.051	3.8e-01
Precuneus	Externalizing	Internalizing, Externalizing & DP	0.013	0.037	7.23e-01
Precuneus	Dysregulation Profile	Internalizing, Externalizing & DP	-0.102	0.042	1.44e-02
Orbitofrontal	Externalizing	Externalizing & DP	-0.001	0.039	9.77e-01
Orbitofrontal	Dysregulation Profile	Externalizing & DP	-0.09	0.055	9.77e-02
Rostral middle frontal	Externalizing	Externalizing & DP	0.048	0.039	2.2e-01
Rostral middle frontal	Dysregulation Profile	Externalizing & DP	-0.18	0.055	1.2e-03
Amygdala	Externalizing	Externalizing & DP	-0.056	0.037	1.25e-01
Amygdala	Dysregulation Profile	Externalizing & DP	-0.025	0.051	6.33e-01
Cuneus	Externalizing	Externalizing & DP	0.07	0.038	6.5e-02
Cuneus	Dysregulation Profile	Externalizing & DP	-0.209	0.053	8.5e-05
Fusiform	Externalizing	Externalizing & DP	-0.022	0.039	5.7e-01
Fusiform	Dysregulation Profile	Externalizing & DP	-0.064	0.056	2.5e-01
Inferior parietal	Externalizing	Externalizing & DP	-0.003	0.039	9.41e-01
Inferior parietal	Dysregulation Profile	Externalizing & DP	-0.117	0.055	3.27e-02
Inferior temporal	Externalizing	Externalizing & DP	0.012	0.039	7.58e-01
Inferior temporal	Dysregulation Profile	Externalizing & DP	-0.102	0.055	6.08e-02
Isthmus cingulate	Externalizing	Externalizing & DP	0.033	0.038	3.97e-01
Isthmus cingulate	Dysregulation Profile	Externalizing & DP	-0.15	0.054	5.69e-03
Lateral occipital	Externalizing	Externalizing & DP	0.047	0.038	2.14e-01
Lateral occipital	Dysregulation Profile	Externalizing & DP	-0.193	0.054	3.47e-04
Middle temporal	Externalizing	Externalizing & DP	0.026	0.039	5.14e-01
Middle temporal	Dysregulation Profile	Externalizing & DP	-0.146	0.055	8.49e-03
Posterior cingulate	Externalizing	Externalizing & DP	0.035	0.038	3.58e-01
Posterior cingulate	Dysregulation Profile	Externalizing & DP	-0.15	0.054	5.83e-03
Precentral	Externalizing	Externalizing & DP	0.022	0.039	5.81e-01
Precentral	Dysregulation Profile	Externalizing & DP	-0.145	0.056	9.3e-03
Superior parietal	Externalizing	Externalizing & DP	-0.012	0.038	7.51e-01
Superior parietal	Dysregulation Profile	Externalizing & DP	-0.069	0.054	2e-01
Thalamus	Externalizing	Externalizing & DP	0.05	0.039	2e-01
Thalamus	Dysregulation Profile	Externalizing & DP	-0.16	0.055	3.6e-03

Table S12. Simultaneously modelled significant psychpathology domains in relation to deviations from typical development for cortical thickness

Brain region	Psychopathology symptoms	Psychopathology symptoms modelled simultaneously	B	S.E.	p-value
Orbitofrontal	Externalizing	Externalizing & DP	0.02	0.027	4.72e-01
Orbitofrontal	Dysregulation Profile	Externalizing & DP	0.02	0.038	5.96e-01
Frontalpole	Externalizing	Externalizing & DP	-0.001	0.032	9.66e-01
Frontalpole	Dysregulation Profile	Externalizing & DP	0.041	0.044	3.55e-01
Posteriorcingulate	Externalizing	Externalizing & DP	0.036	0.033	2.79e-01
Posteriorcingulate	Dysregulation Profile	Externalizing & DP	0.018	0.046	6.91e-01

Table S13. Hypothesis driven surface area model 1

Brain region	Psychopathology symptoms	B	S.E.	p-value
Anterior cingulate	Internalizing	-0.358	0.082	1.22e-05
	Externalizing	-0.449	0.082	4.6e-08
	Dysregulation Profile	-0.703	0.113	7.08e-10
Orbitofrontal	Internalizing	-0.229	0.08	4.16e-03
	Externalizing	-0.39	0.08	1.2e-06
	Dysregulation Profile	-0.541	0.109	7.84e-07
Rostral middle frontal	Internalizing	-0.334	0.082	4.91e-05
	Externalizing	-0.437	0.082	1.13e-07
	Dysregulation Profile	-0.67	0.113	3.94e-09

Table S14. Exploratory surface area model 1

Brain region	B	S.E.	p-value	B	S.E.	p-value	B	S.E.	p-value
Bankssts	-0.169	0.085	4.84e-02	-0.336	0.086	8.98e-05	-0.457	0.119	1.2e-04
Caudal middle frontal	-0.281	0.082	6.55e-04	-0.309	0.082	1.8e-04	-0.517	0.114	6.58e-06
Cuneus	-0.427	0.082	2.46e-07	-0.472	0.083	1.35e-08	-0.679	0.114	3.63e-09
Entorhinal	-0.032	0.082	6.97e-01	-0.09	0.083	2.77e-01	-0.11	0.113	3.3e-01
Frontal pole	-0.219	0.077	4.58e-03	-0.329	0.078	2.29e-05	-0.442	0.105	2.57e-05
Fusiform	-0.251	0.082	2.13e-03	-0.391	0.082	1.97e-06	-0.554	0.113	1.14e-06
Inferior parietal	-0.289	0.083	5.42e-04	-0.451	0.083	6.89e-08	-0.592	0.116	3.53e-07
Inferior temporal	-0.294	0.081	2.78e-04	-0.455	0.081	2.03e-08	-0.589	0.111	1.45e-07
Insula	-0.15	0.082	6.93e-02	-0.222	0.083	7.26e-03	-0.448	0.113	7.56e-05
Isthmus cingulate	-0.298	0.087	6.08e-04	-0.414	0.087	1.95e-06	-0.619	0.121	3.2e-07
Lateral occipital	-0.528	0.081	9.54e-11	-0.552	0.081	1.52e-11	-0.748	0.113	4.28e-11
Lingual	-0.317	0.082	1.21e-04	-0.462	0.082	2.29e-08	-0.631	0.114	3.76e-08
Middle temporal	-0.294	0.082	3.3e-04	-0.476	0.082	7.17e-09	-0.644	0.113	1.45e-08
Paracentral	-0.137	0.085	1.06e-01	-0.317	0.085	2.01e-04	-0.413	0.118	4.88e-04
Parahippocampal	0.053	0.082	5.15e-01	-0.199	0.082	1.55e-02	-0.134	0.113	2.39e-01
Pars opercularis	-0.225	0.085	8.01e-03	-0.315	0.085	2.15e-04	-0.522	0.118	1.03e-05
Pars orbitalis	-0.278	0.081	6.75e-04	-0.431	0.082	1.48e-07	-0.567	0.112	5.05e-07
Pars triangularis	-0.278	0.083	8.39e-04	-0.383	0.083	4.28e-06	-0.611	0.115	1.27e-07
Pericalcarine	-0.297	0.084	4.23e-04	-0.372	0.084	1.04e-05	-0.561	0.116	1.58e-06
Posterior cingulate	-0.242	0.085	4.32e-03	-0.421	0.085	7.46e-07	-0.655	0.118	2.99e-08
Postcentral	-0.308	0.086	3.52e-04	-0.33	0.086	1.34e-04	-0.555	0.119	3.68e-06
Precentral	-0.332	0.083	7.44e-05	-0.476	0.083	1.43e-08	-0.688	0.116	3.39e-09
Precuneus	-0.345	0.085	4.97e-05	-0.456	0.085	8.67e-08	-0.633	0.118	9.13e-08
Superior frontal	-0.228	0.082	5.22e-03	-0.334	0.082	4.57e-05	-0.505	0.113	8.67e-06
Superior parietal	-0.35	0.081	1.79e-05	-0.388	0.081	2.02e-06	-0.478	0.113	2.38e-05
Superior temporal	-0.248	0.085	3.72e-03	-0.419	0.085	1.01e-06	-0.667	0.118	2.08e-08
Supramarginal	-0.252	0.086	3.5e-03	-0.285	0.086	9.94e-04	-0.458	0.12	1.44e-04
Temporal pole	-0.14	0.077	6.93e-02	-0.226	0.077	3.56e-03	-0.301	0.104	3.91e-03
Transverse temporal	-0.189	0.087	2.97e-02	-0.296	0.087	6.76e-04	-0.506	0.121	3.12e-05

2.2 Figures

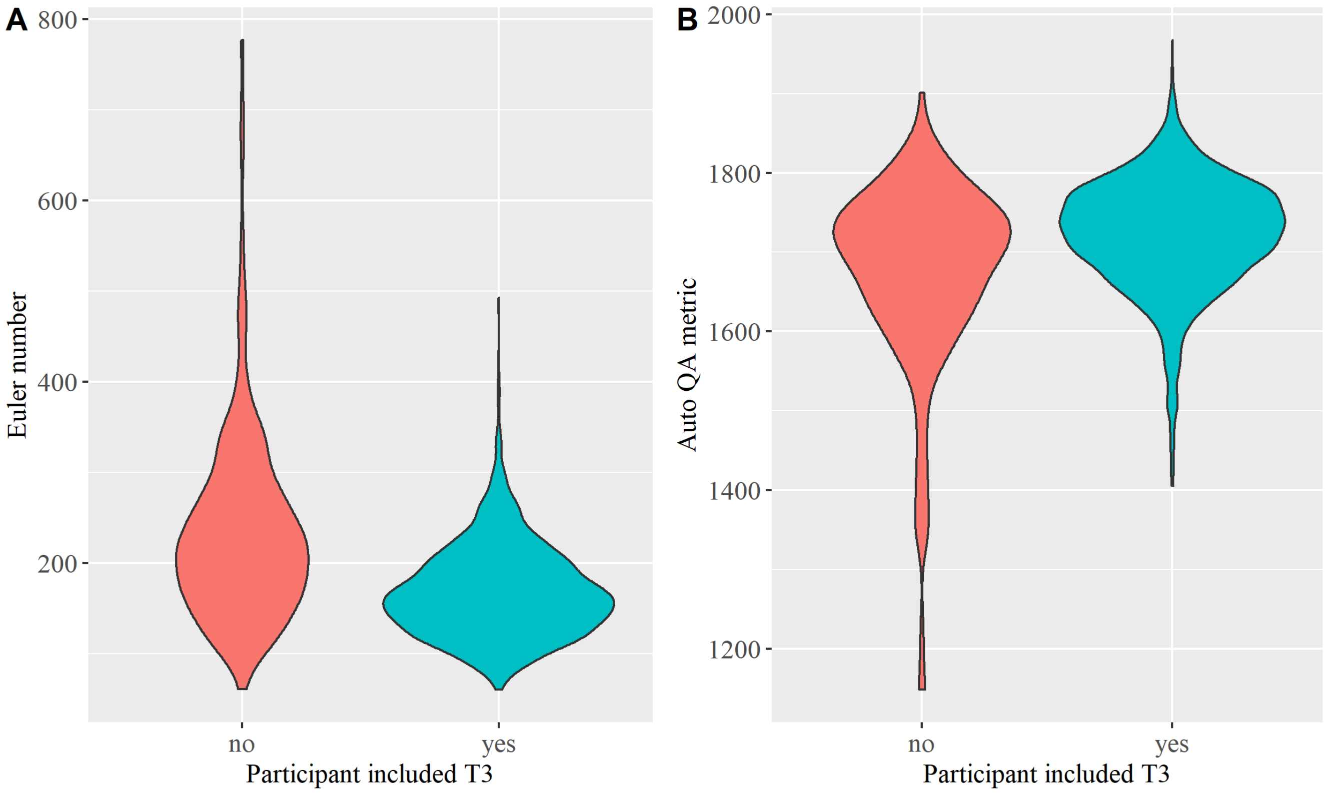

Figure S1. Automated quality assessment for included and excluded scans based on visual inspection; (A) represents the Euler number and (B) the automated quality assessment described in White et al. (2018).

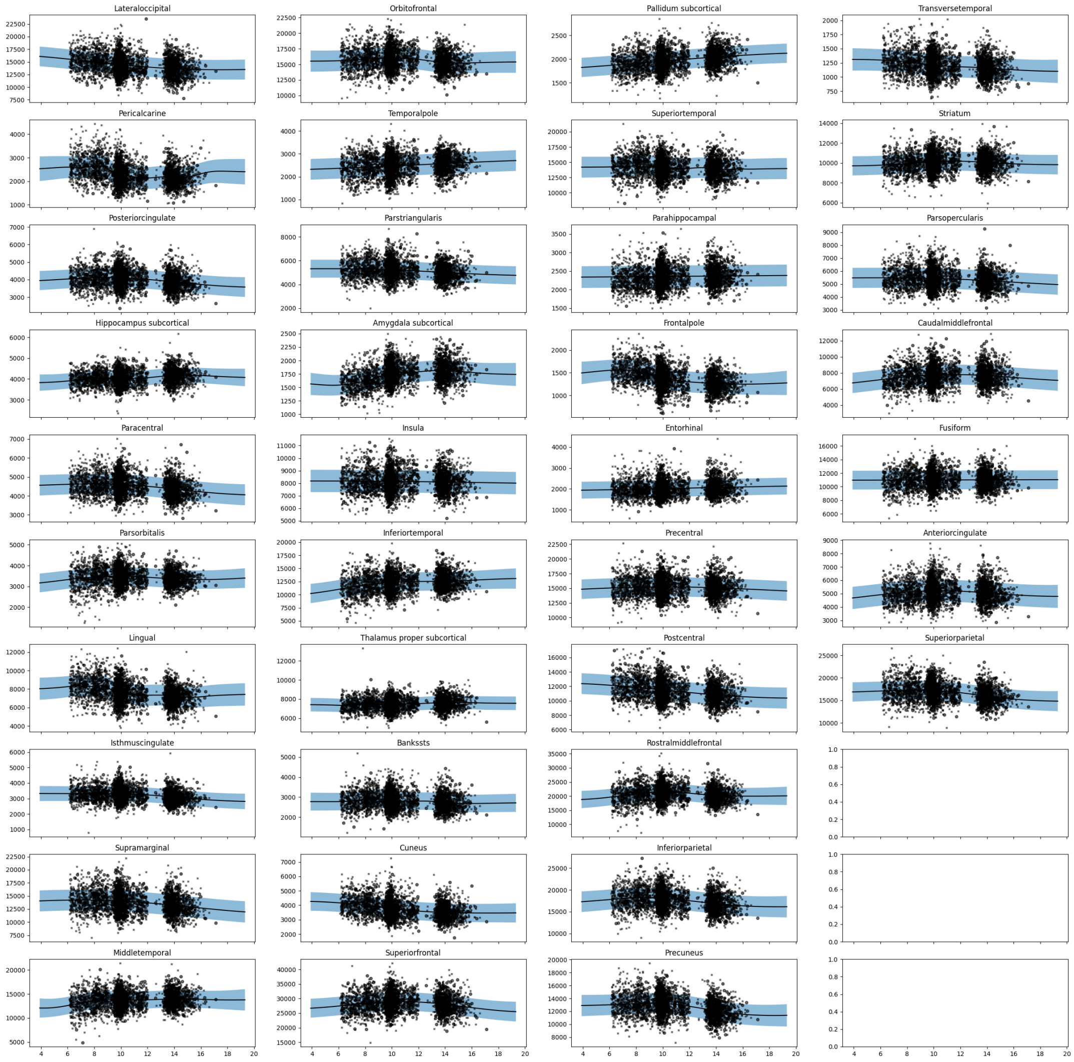

Figure S2. The (sub-)cortical volume for each subject in test fold and the fit of the normative model to the (sub-)cortical volume in the training set. Predicted normative model is presented in a wider age range than the data that was included to derive predictions. For illustrative purposes, measures are rescaled to the original means and standard deviations.

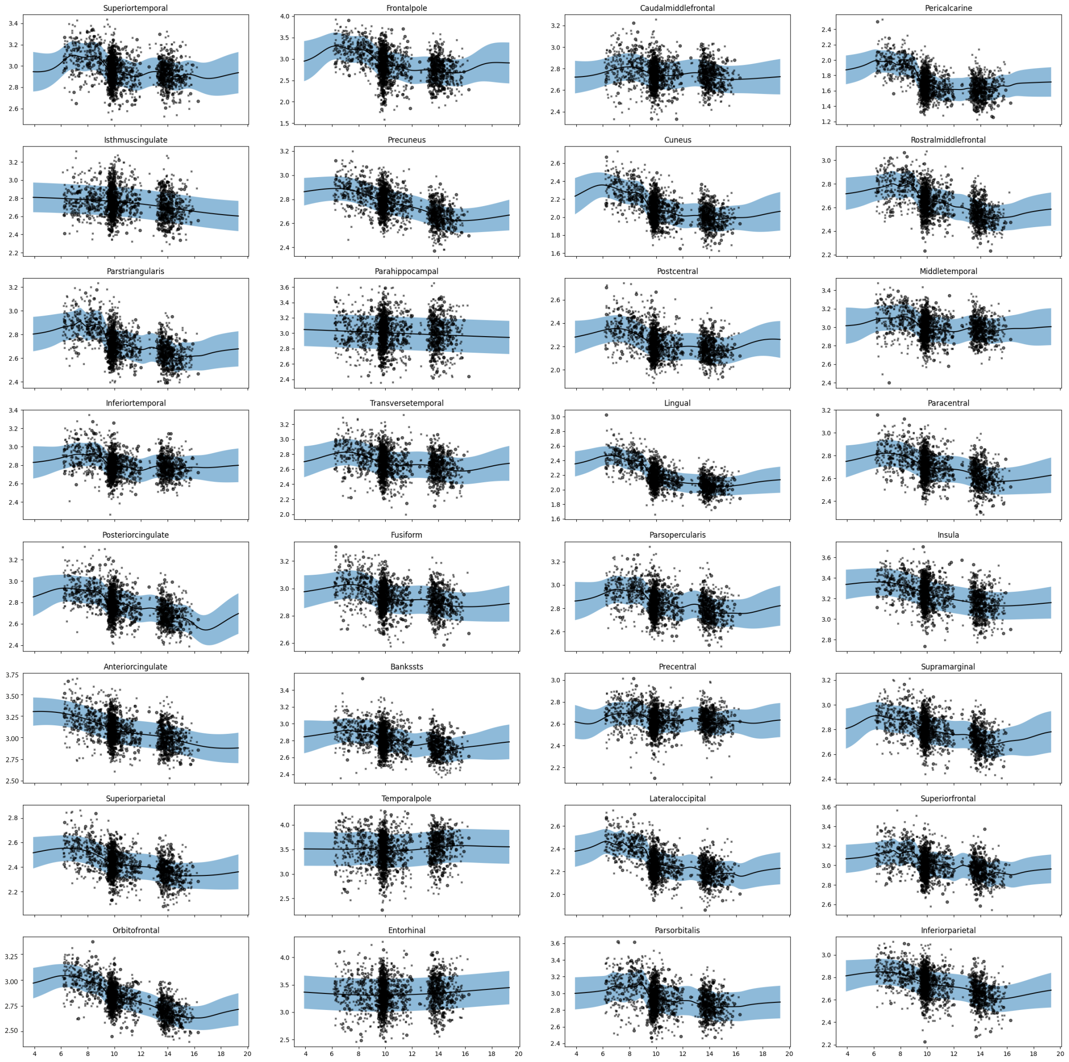

Figure S3. The cortical thickness for each subject in test fold and the fit of the normative model to the cortical thickness in the training set. Predicted normative model is presented in a wider age range than the data that was included to derive predictions. For illustrative purposes, measures are rescaled to the original means and standard deviations.

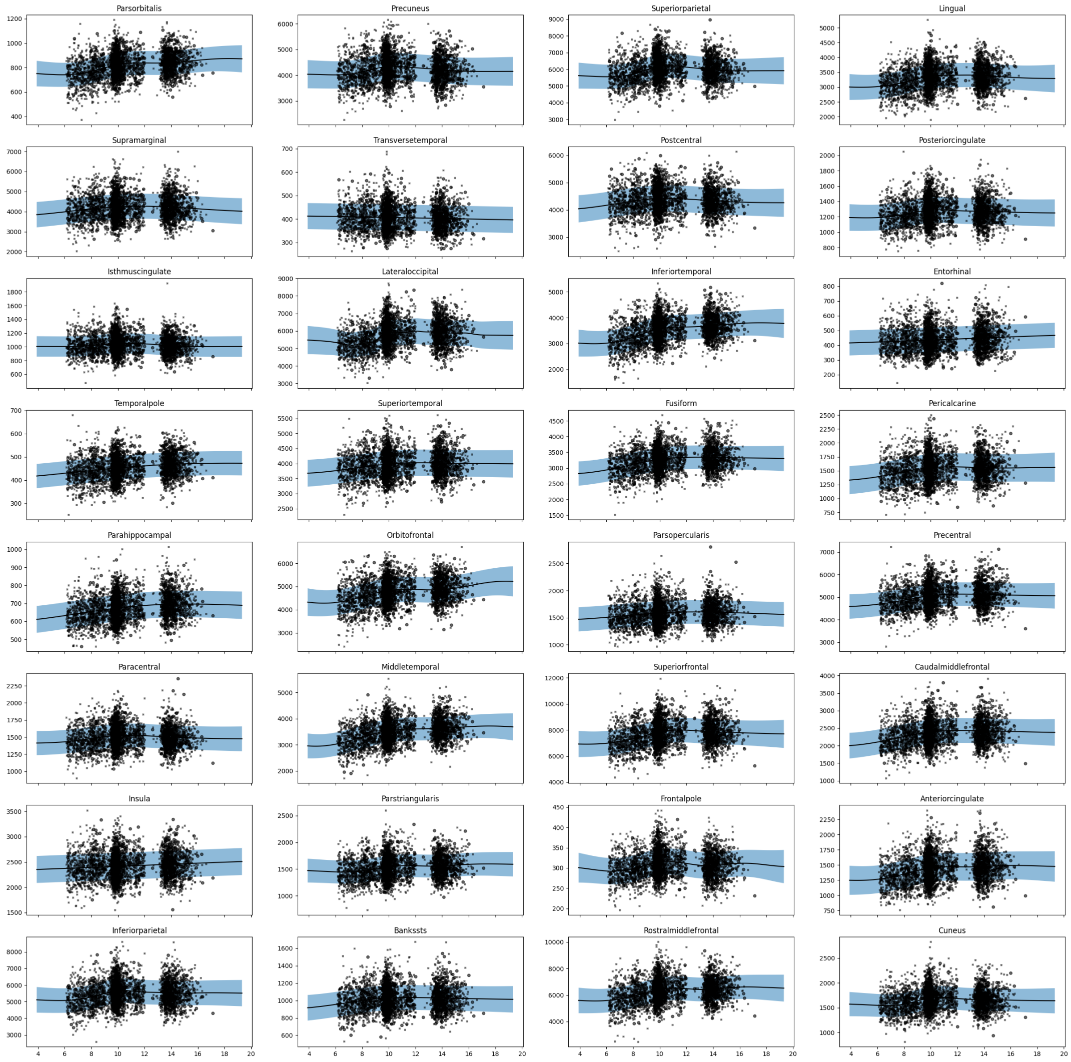

Figure S4. The surface area for each subject in test fold and the fit of the normative model to the surface area in the training set. Predicted normative model is presented in a wider age range than the data that was included to derive predictions. For illustrative purposes, measures are rescaled to the original means and standard deviations.
